# Species and Population Level Molecular Profiling Reveals Cryptic Recombination and Emergent Asymmetry in the Dimorphic Mating Locus of *C. reinhardtii*

**DOI:** 10.1371/journal.pgen.1003724

**Published:** 2013-08-29

**Authors:** Peter L. De Hoff, Patrick Ferris, Bradley J. S. C. Olson, Ayano Miyagi, Sa Geng, James G. Umen

**Affiliations:** 1 The Salk Institute for Biological Studies, La Jolla, California, United States of America; 2 Department of Ecology and Evolutionary Biology, University of Arizona, Tucson, Arizona, United States of America; 3 Division of Biology, Kansas State University, Manhattan, Kansas, United States of America; 4 Donald Danforth Plant Science Center, St. Louis, Missouri, United States of America; Duke University Medical Center, United States of America

## Abstract

Heteromorphic sex-determining regions or mating-type loci can contain large regions of non-recombining sequence where selection operates under different constraints than in freely recombining autosomal regions. Detailed studies of these non-recombining regions can provide insights into how genes are gained and lost, and how genetic isolation is maintained between mating haplotypes or sex chromosomes. The *Chlamydomonas reinhardtii* mating-type locus (*MT*) is a complex polygenic region characterized by sequence rearrangements and suppressed recombination between its two haplotypes, *MT+* and *MT−*. We used new sequence information to redefine the genetic contents of *MT* and found repeated translocations from autosomes as well as sexually controlled expression patterns for several newly identified genes. We examined sequence diversity of *MT* genes from wild isolates of *C. reinhardtii* to investigate the impacts of recombination suppression. Our population data revealed two previously unreported types of genetic exchange in *Chlamydomonas MT*—gene conversion in the rearranged domains, and crossover exchanges in flanking domains—both of which contribute to maintenance of genetic homogeneity between haplotypes. To investigate the cause of blocked recombination in *MT* we assessed recombination rates in crosses where the parents were homozygous at *MT*. While normal recombination was restored in *MT+*×*MT+* crosses, it was still suppressed in *MT−*×*MT−* crosses. These data revealed an underlying asymmetry in the two *MT* haplotypes and suggest that sequence rearrangements are insufficient to fully account for recombination suppression. Together our findings reveal new evolutionary dynamics for mating loci and have implications for the evolution of heteromorphic sex chromosomes and other non-recombining genomic regions.

## Author Summary

Sex chromosomes and mating-type loci are often atypical in their structure and evolutionary dynamics. One distinguishing feature is the absence of recombination that results in genetic isolation and promotes rapid evolution and sometimes degeneration. We investigated gene content, sex-regulated expression, and recombination of mating locus (*MT*) genes in the unicellular alga *Chlamydomonas reinhardtii*. Despite the lack of observable recombination in and around *Chlamydomonas MT*, genes from its two mating types are far more similar to each other than expected for a non-recombining region. This discrepancy is explained by our finding evidence of genetic exchange between the two mating types within wild populations. In addition, we observed an unexpected asymmetry in the recombination behavior of the two mating types that may have contributed to the preferential expansion of one *MT* haplotype over the other through insertion of new genes. Our data suggest a mechanism to explain the emergence of heteromorphic sex chromosomes in haploid organisms by asymmetric expansion rather than by loss or degeneration as occurs in some Y or W chromosomes from diploid organisms. Our observations support a revised view of recombination in sex-determining regions as a quantitative phenomenon that can significantly affect rates of evolution and sex-linked genetic diversification.

## Introduction

Heteromorphic sex chromosomes and mating-type loci can be dynamic genomic regions with large non-recombining blocks of rearranged sequences, high transposon and repeat density, low-protein coding gene density, and high rates of sequence evolution compared to autosomes [1]–[3]. Sex chromosomes undergo decay and gene loss [4], but have also been found to be sources of genetic innovation [5]. Sex determining or mating-type regions in haploid species are diverse and can be controlled by small mating-type loci with one or two genes, as in the case of yeasts [6], by complex heteromorphic mating-type loci such as those found and algae and some fungi [7]–[11], or by sex chromosomes in bryophytes [12], [13].

Volvocine algae are an emerging model for investigating the evolution of sex chromosomes and mating-type loci [14]. These haploid green algae form a coherent phylogenetic group that encompasses unicellular species such as *Chlamydomonas reinhardtii* and multicellular species such as *Volvox carteri*. Volvocine algae show convergent evolution with other multicellular clades in their sexual cycles: isogamy (equal-sized gametes) is predominant in small colonial genera and unicellular species such as *Chlamydomonas*, while anisogamy (large and small gametes) or oogamy (eggs and sperm) are predominant in larger colonial genera such as *Volvox, Pleodorina* and *Eudorina*. Homothallic and heterothallic mating systems also evolved within different Volvocine algal sub-lineages making them a highly diverse group [15]–[17].

*Chlamydomonas reinhardtii* is a heterothallic species with two mating types, *plus* (*MT+*) and *minus* (*MT−*), which are defined by alleles at its mating locus (*MT*) located near one telomere of Chromosome 6. Haploid cells of either mating type can propagate mitotically when supplied with sufficient light and nutrients, but differentiate into mating-competent gametes in the absence of nitrogen. Gametes of opposite mating type recognize each other and fuse to form dormant diploid zygospores. When returned to light and nutrients zygospores undergo meiosis to produce two *MT+* and two *MT−* progeny that reenter the vegetative mitotic reproductive cycle (Figure S1).

While *MT* segregates as a single Mendelian trait, it is a genetically complex region encompassing around 200–400 kb of sequence that is rearranged between the two mating-type haplotypes. This rearranged region (R-domain) is flanked by telomere proximal (T) and centromere-proximal (C) domains that are collinear between the mating types, but where recombination is also suppressed [18].

Within the R-domain of *MT* are *sex-limited* genes (present in only one of the two mating haplotypes, *MT+ or MT−*) that are involved in sex determination and other aspects of the sexual cycle. However most of the genes in the R domain are *shared* genes with alleles present in both *MT+* and *MT−* that are arranged in different relative order and/or orientation between the two haplotypes.

A previous study of the *MT* genes and their expression patterns was done before either haplotype was sequenced. Restriction fragment probe hybridization to Northern blots revealed both sex-regulated and constitutively-expressed genes within *MT*, but was limited to finding well-expressed genes with favorable hybridization characteristics [19]. More recent sequencing of the full genome of a *MT+* strain, and of the *MT−* haplotype allowed a more comprehensive identification and prediction of *Chlamydomonas MT* genes [11], [20]. However, gene model validation and expression patterns for many of these genes have not been previously reported.

Although sex-linked polymorphisms are evident between *MT+* and *MT−* alleles of genes in the R, C and T domains, the degree of haplotype differentiation in *Chlamydomonas* is unexpectedly low when compared to the male and female *MT* haplotypes of *Volvox carteri* that are physically much larger (>1 Mb), but derived from a region of *Volvox* linkage group I that is syntenic with *Chlamydomonas MT* and chromosome 6 [11]. Assuming no recombination occurred within *MT* for either species, it is expected that the *MT+* and *MT−* alleles for genes in *Chlamydomonas* would be at least as diverged as those from *Volvox* female and male *MT* haplotypes [14], [21], but this is not the case: *Volvox MT* neutral divergence levels are about 100-fold higher than those for *Chlamydomonas MT* genes. This divergence paradox might be explained if rare recombination or genetic exchange occurred between *MT+* and *MT−* genes of *Chlamydomonas* [14].

A second unexplained difference between *MT* of *Chlamydomonas* and *MT* of *Volvox* is the rates of recombination observed in their C and T domains that are the collinear regions immediately proximal to either side of the R domain of *MT*. In *Chlamydomonas* the C/T regions show suppressed recombination over several hundred kb, whereas in *Volvox*, crossovers were observed <30 kb from the R-domain [11]. Thus, proximity to a large rearranged region appears to be insufficient to explain suppressed recombination in nearby flanking collinear regions. The causes of different recombination behaviors between the *Chlamydomonas* and *Volvox MT* regions have not been previously investigated.

Here we examined gene content and expression of *Chlamydomonas MT* genes in greater detail than previously possible. Our investigations revealed new R-domain sequences caused by translocations into the *MT+* locus bringing the total of such events to three and substantially increasing the size of the *MT+* R-domain. We validated expression for 29 *MT* gene models and found sex-regulated expression patterns for a subset of uncharacterized *MT* genes. In addition we used population genetic data for *Chlamydomonas MT* genes to reassess their history of genetic exchange and potential for recombination. These experiments revealed a history of gene conversion in the R-domain as well as genetic exchange in the C and T domains. Finally, we examined the recombination potential of *MT* genes by performing crosses where each of the parents contained the same *MT* haplotype (*MT+*×*MT+* or *MT−*×*MT*). These crosses revealed an underlying asymmetry between *MT+* and *MT−* and suggest the presence of sequences in *MT−* that repress recombination in *MT* even when a collinear partner is available for meiotic pairing.

## Results

Our results are divided into four sections. First, we describe new structural features of the *C. reinhardtii* mating locus revealed from sequencing both haplotypes. Second, we describe sexually controlled expression patterns of newly-described mating locus genes. Third, we use population genetics to identify rare genetic exchange events between *MT* haplotypes. Finally, we examine the potential for recombination in *MT* in crosses engineered so that both *MT* haplotypes are identical and collinear.

### Revised description of structure and genetic content for the *C. reinhardtii MT* locus

**Figure 1:**
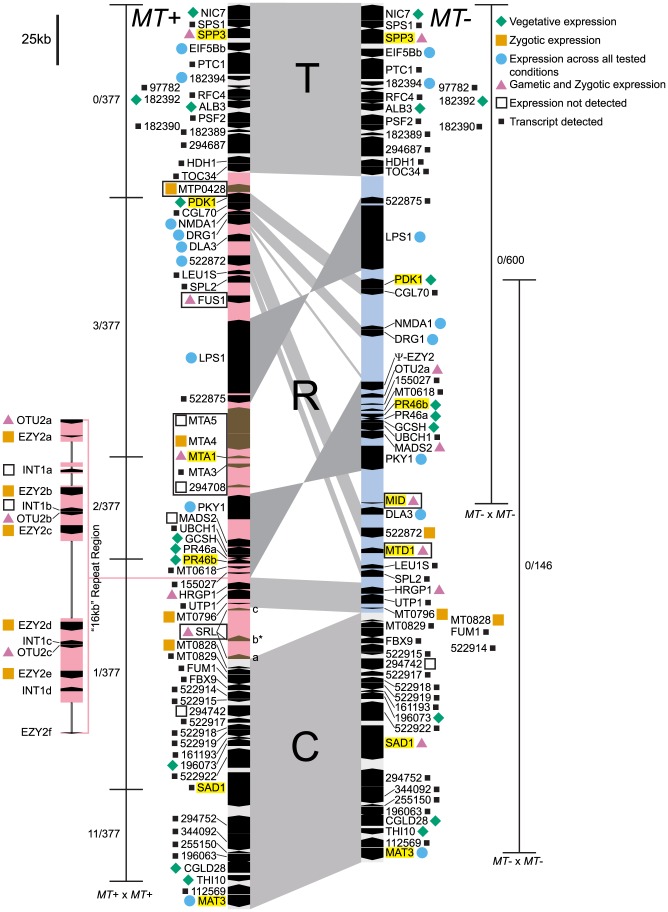
Diagram of the *Chlamydomonas reinhardtii* mating locus. The *MT+* (left side) and *MT−* (right side) haplotypes are aligned vertically with regions of synteny connected by gray shading. The three major domains are labeled as T, (Telomere Proximal, ∼82–84 kb), R (Rearranged, ∼204–396 kb), and C (Centromere Proximal, ∼116 kb). The R-domain section of each haplotype is shaded light pink (*MT+*) or blue (*MT−*). Genes are designated by black or brown pointed rectangles with pointed ends showing their relative orientation. Gene names are shown to the left or right of each gene symbol. The 16 kb repeat region in *MT+* is depicted as an expansion to the left of the main diagram with unassembled regions indicated by thin lines. *MT+* and *MT−* limited genes are boxed. Names of genes used for population studies are highlighted in yellow. Gene expression patterns compiled from this study, from [19], and from publicly available transcriptome data are denoted by colored shapes as follows: blue circle, all stages; green diamond, vegetative; pink triangle, gametic and zygotic; orange square, zygotic; open square, not detected; small black square, transcript detected but expression pattern not determined. The expression pattern shown for the *SRL* region is specific to the *SRLb* gene that is indicated by an asterisk. The thin bars to the left and right of each diagram show the region where recombination was measured in *MT+*×*MT+* or *MT−*×*MT−* homozygous crosses. Crosshatches show markers that were scored for recombination and numbers of recombinants/total progeny scored are shown next to each recombination interval.

Structural data on the *Chlamydomonas reinhardtii* mating locus (hereafter referred to as *Chlamydomonas MT*) was previously based on a restriction-enzyme-mapped phage walk through both mating types [22]. In addition, the published V3 genome sequence contains portions of the *plus* haplotype (*MT+*) but its assembly was not contiguous through the mating locus [20]. An updated assembly of Chromosome 6 available through Phytozome [23] is contiguous through the *MT+* region, though there are still some repeats whose copy number has not been accurately determined. We recently cloned and sequenced the *minus* haplotype (*MT−*) that allowed direct comparisons between nearly complete sequences of both mating types from *Chlamydomonas* (Figure 1 and [11]). Below we describe new and updated analyses of *Chlamydomonas MT* including two regions of the *MT+* haplotype that derive from autosomal insertions, a redefined border for the R-domain, and a revised description of the 16 kb repeat region.

#### Autosomal insertions into *MT*

**Figure 2:**
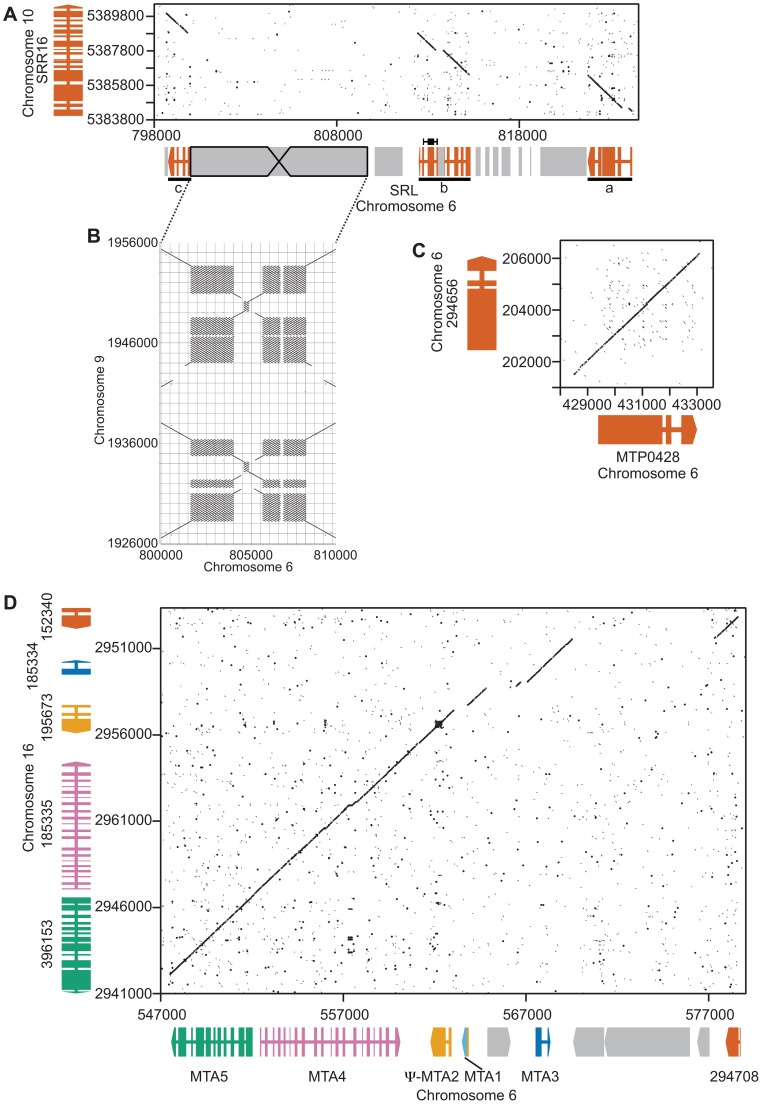
Structure of three *MT+* regions derived from autosomal duplications. Dot plot comparisons of mating locus and autosomal regions. A. Autosomal gene *SRR16* (y axis) and the *SRL* region (x axis). Wide and narrow colored rectangles depict exons and introns respectively for *SRR16* and the *SRL* region that is broken into three underlined segments—a, b and c. Gray shaded regions represent repeats and transposons. B. Structure of a large inverted repeat within *SRL* that derives from chromosome 9. C. Autosomal gene *294656* (y axis) with *MTP0428* (x axis). Gene structures are as described in Panel A. D. Autosomal **a** region (x axis) and *MTA* region (y axis). Individual genes are different colors with gene structures depicted as in Panel A.

SRL *region*. Similarity searches done with *MT* sequences queried against autosomes revealed a domain of *MT+* that we termed the *SRL* region whose discovery extends the R-domain by ∼30 kb (Figure 1). *SRL* arose through duplication-insertion of a ∼5.7 kb segment of *SRR16* from Chromosome 10 into the *MT+* locus (Figure 2A, Table S1). The full-length *SRR16* gene encompasses ∼60 kb and encodes a predicted transmembrane scavenger receptor protein of 797 kDa with two scavenger receptor (SR) like domains followed by a glycosyl hydrolase (GH) domain and fourteen C-type lectin (CTL) domains [24], none of which are present in the translocated *SRL* region. Further analyses of the *SRL* region showed that additional rearrangements and secondary insertions took place after it moved into the mating locus (Figure 2A). These secondary insertions divided the *SRR16*-homologous region into three large blocks that are designated *SRLa*, *SRLb* and *SRLc*. The largest secondary insertion into *SRL* comes from Chromosome 9 and derives from an uncharacterized segmental repeat that has undergone at least two cycles of duplication-inversion (Figure 2B).

##### *MTP0428* gene:

The telomere proximal border of the *MT+* R-domain (previously described as region b in [19]), contains an uncharacterized gene, *MTP0428*, that has a full length duplicate copy and two partial copies on autosomal portions of Chromosome 6 (Figures 1, 2C, and Table S1). The predicted MTP0428 protein has no identifiable domains and no identifiable homologs outside of *Chlamydomonas*.

##### *MTA* region:

The mating-type *a* region (*MTA*) found in the *MT+* haplotype was previously described and found to be derived from an autosomal translocation [19]. Here we identify its source as a ∼25 kb contiguous portion of Chromosome 16 that inserted between the *MT+* genes *522875* and *PKY1* (Figure 1). The *MTA* region contains three full-length genes from Chromosome 16—*MTA2*, *MTA3* and *MTA4*—and two partially duplicated genes—*294708* and *MTA5*—whose autosomal homologs straddle the translocation breakpoints (Figure 2D, Table S1). *MTA2* was subsequently modified by insertion of sequences from Chromosome 7 into its first exon to generate a chimeric gene, *MTA1*, while the downstream exons of *MTA2* became a pseudogene [19]. *MTA4* acquired a premature stop codon mutation about half way through its coding region relative to its autosomal counterpart. All of the *MTA*/Chromosome 16 genes in the translocated region encode proteins that are lineage specific: MTA2/195673 is a putative hydroxyproline-rich glycoprotein (HRGP) with no homologs outside of *Chlamydomonas*, while the remaining encoded proteins have autosomal homologs in *Volvox carteri* but nowhere outside of Volvocine algae (data not shown).

#### Divergence of autosomally-derived *MT* genes

**Figure 3:**
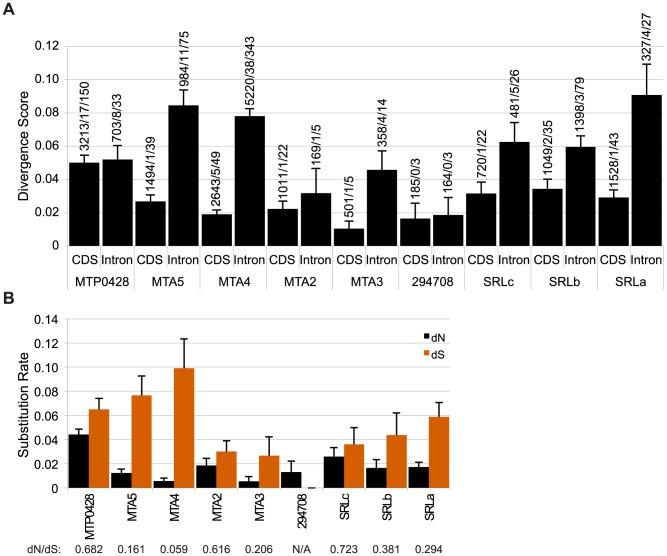
Divergence of autosomal and *MT+* duplicated genes. A. Bar graph of nucleotide divergence values [77] for alignments of coding (CDS) and intron sequences of *MT+* genes and their autosomal progenitors as shown in Figure 2. Bars depict divergence with standard error indicated by lines. Values above each bar show total number of aligned bases/number of indels/number of substitutions for the alignment. B. Synonymous (dS) and non-synonymous (dN) substitution rates for CDS alignments described in Panel A with the standard error indicated by the line on top of each bar. dN/dS ratios are shown below each gene.

We expected that the *MTP0428*, *MTA* and *SRL* regions might behave as “strata” [25], [26] with neutral divergence correlated with the timing of each separate insertion/duplication event as has been proposed for mating-type chromosomes in other systems [8], [27]–[29]. Intron divergence was used as a metric for neutral rates of evolution, but we also examined intergenic regions in *MTA* and silent substitutions in coding regions for all three duplicated segments (Figure 3, Table S2). The neutral divergence patterns of the *MTA* region were highly variable. On one end of the *MTA* region is *294708* that has a relatively low intronic divergence value of 0.0186 (98% alignment identity), while on the other end is *MTA5* with an intronic divergence value of 0.0844 (89% alignment identity). *MTA4* shows a similar pattern as *MTA5* while *MTA2* and *MTA3* are in between. dS values for coding regions followed a similar pattern as intronic divergence (Figure 3B) while intergenic divergence was less variable (∼0.5–0.8) (Table S2). The divergence data for *MTP0428* and the *SRL* region are not sufficiently different from the *MTA* region that we can assign a relative time to their insertions into *MT+* (Figure 3, Table S2).

The ratio of synonymous (dS) and non-synonymous (dN) substitution rates within coding sequences provide a measure of the strength of selection on one or both duplicate copies of a gene. Low dN/dS ratios imply strong purifying selection on both copies as is seen for MTA4/185335 with a value of 0.059 (Figure 3B, Table S2). Other duplicate genes such as MTP0428/294656 and SRLc/SRR16 have higher dN/dS ratios of 0.682 and 0.723 respectively. Our data cannot determine whether one or both genes in the duplicate pair are under positive selection or are evolving neutrally as the dN/dS ratios indicate. Codon adaptation indices (CAI) [30] can provide an indirect measure of differing selection on homologs [31], but we found no significant differences in CAI or codon mutational bias for *MT+* versus autosomal paralogs in this study (Table S3 and data not shown).

#### 16 kb repeat region

**Figure 4:**
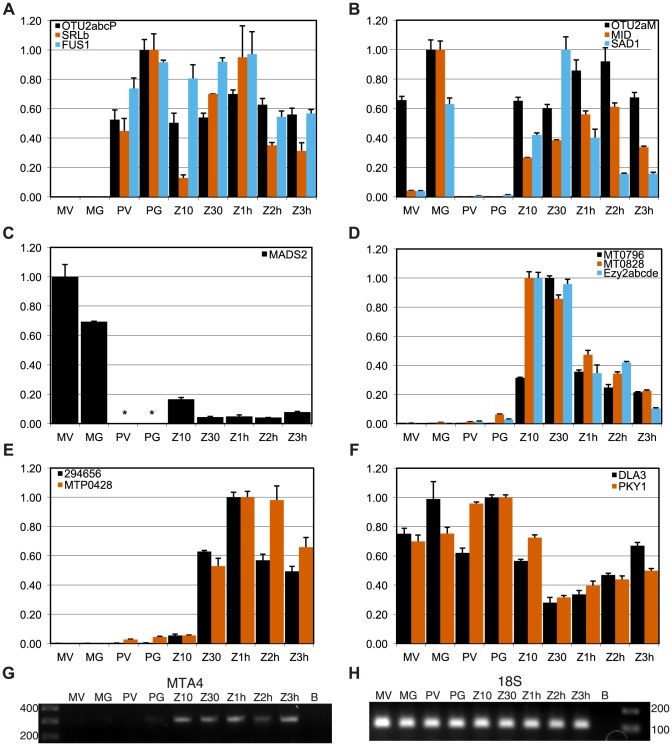
Expression patterns of mating locus genes. Panels A–F show expression values from quantitative RT-PCR (qRT-PCR) experiments for indicated genes calculated as described in Materials and Methods. Each panel groups genes by their overall expression pattern as follows: A, *MT+* gametic; B, *MT−* gametic; C, *MT−* only; D, early zygotic; E, zygotic; F, reduced in zygotes. RNA samples were derived from *MT+* vegetative cells (PV) and gametes (PG), *MT−* vegetative cells (MV) and gametes (MG), and from zygotes at 10 minutes, 30 minutes, 1 hour, 2 hours and 3 hours after mating (Z10, Z30, Z1h, Z2h and Z3h respectively). Panels G, H show gels from semi-quantitative RT-PCR experiments in which G. *MTA4* cDNA or H. internal control 18S ribosomal cDNA were amplified. * No expression detected.

A ∼160 kb region of *MT+* Chromosome 6 consists of around nine or ten copies of a ∼17 kb (17,217 bp) tandem repeat termed the “16 kb repeats” in [19]. At least three genes are found within the 16 kb repeats: *EZY2* encodes a predicted chloroplast protein with no recognizable domains or similarity, and its mRNA is zygote specific [19] (Figure 4D). There are at least six copies of *EZY2* in the 16 kb repeat region (Figure 1, Table S4) designated *EZY2a-EZY2f* and a single *EZY2* pseudogene in the *MT−* locus (Figure 1). Based on its presence in *MT+*, its zygotic expression pattern, and predicted chloroplast localization *EZY2* was proposed to be involved in uniparental chloroplast DNA inheritance [18], [19]. *OTU2* encodes a putative otubain-related protease [18]. The three copies of *OTU2* that could be distinguished based on polymorphisms are designated *OTU2a-OTU2c*. A single copy of *OTU2a* that resides in the *MT−* R-domain (Figure 1) was not previously described. *INT1* encodes a putative retroviral-related integrase that is present in some of the 16 kb repeats but nowhere else in the *Chlamydomonas* genome (Figure 1 and Table S4). The open reading frame of the *INT1* gene contains a frame-shift mutation that would prevent production of a full-length polypeptide in the absence translational frame shifting; however, we were unable to detect any mRNA corresponding to *INT1* (Figure 1 and data not shown).

#### Shared genes in *MT*

The *MT* locus contains sex-limited genes (e.g. *MID* in *MT−* and *FUS1* in *MT+*), as well as shared genes that have an allele in both mating types (Figure 1). A few shared genes in the rearranged domain of *MT* encode enzymes involved in primary metabolism such as *PDK1* (pyruvate dehydrogenase kinase), *GCSH* (glycine decarboxylase subunit H), *LEU1S* (isopropylmalate dehydratase, small subunit) and *DLA3* (dihydrolipoamide acetyltransferase). There are also a pair of convergently transcribed genes with overlapping 3′ untranslated regions, *PR46a* and *PR46b*, whose configuration and putative protein products are conserved in diverse eukaryotes, including humans, but whose function is not known ([18], [19] and data not shown). Additional shared R-domain genes in *Chlamydomonas* encode conserved proteins of unknown function (*NMDA1, CGL70*), possible signaling proteins including a kinase (*PKY1*), GTP binding protein (*DRG1*), and ubiquitin hydrolase (*UBCH1*), a MADS box transcription factor (*MADS2*), a putative cell wall protein (*HRGP1*), a splicing factor (*SPL2*), and nucleolar protein (*UTP1*). Many of the shared genes in *Chlamydomonas MT* have homologs in or near *Volvox MT* [11], but several do not, including *DLA3*, as well as four genes that encode putative proteins of unknown function, *155027*, *522875*, *MT0796* and *MT0828* (Figure 1 and Table S4).

### Expression patterns of mating locus genes

We determined the expression patterns of selected *MT* genes from vegetative, gametic, and early zygotic RNA samples in order to identify those with possible roles in the sexual cycle. Results of our expression studies and summaries of previous such studies are presented in Figures 1, 4, S4, S5, and Table S5.

#### Sex-limited genes

We used quantitative RT-PCR (qRT-PCR) to determine expression patterns during the sexual cycle of uncharacterized *MT+* genes along with controls that included *SAD1* and *MID* (*minus* gametic expression), *FUS1* (*plus* gametic expression) and *EZY2* (zygotic expression) [18], [19]. Expression of *MTP0428* and its autosomal counterpart, *294656* were both detected in zygotic samples using primers specific for each copy (Figure 4E). *MTA2* and *MTA3* are probable pseudogenes [18], [19], but expression of *MTA4* and *MTA5* has not been tested. *MTA4* transcript was detected using primers that could not amplify its autosomal paralog, and was expressed in *MT+* gametes and zygotes (Figures 4G and S4). Primers specific to the *MT+* copies or to the single *MT−* copy of *OTU2* were used to discriminate expression from each mating-type. *OTU2* from both haplotypes showed similar patterns of strong gametic and weaker zygotic expression (Figure 4AB, Table S5), but total expression from *MT+* was stronger than from *MT−* probably due to the presence of multiple copies of *OTU2* in *MT+* versus a single copy in *MT−* (Figures 1 and S4A). Each of the three *SRL* genes has the potential to generate an mRNA with an in-frame coding sequence (Figure 2A). We were not able to detect expression of *SRLa* and *SRLc*, but we did detect an *SRLb* mRNA whose transcript showed modest up-regulation in gametic and zygotic stages of the life cycle (Figure 4A, Table S5). *SRLb* mRNA was also detected in pooled samples from the *Chlamydomonas* sexual cycle that were subjected to 454 transcriptome sequencing (Figure 2A and [32]).

#### Shared genes

Most of the shared genes in *MT* are expressed constitutively and are presumed to have functions that are not sex-related. *PKY1*, *LPS1* and *DLA3* fall into this category (Figures 4F, S5F, and Table S5).

However, several shared R-domain genes were found to have sex-regulated expression patterns. The putative MADS-box transcription-factor-encoding gene *MADS2* was detected in *MT−* cells from vegetative and gametic samples as well as in zygotes, but not in *MT+* cells (Figures 4C, S5F, and Table S5). PCR primers for detecting *MADS2* cDNA match both *MT+* and *MT−* alleles perfectly, so the expression difference between *MT+* and *MT−* strains is due to mating-type-specific differences that could be cis or trans effects. Inspection of the aligned *MADS2* sequences revealed a point mutation and two indels of 18 bp and 6 bp in its first intron, as well as several polymorphisms and an indel upstream of the start codon (Figure S2). Primers were designed to discriminate between the *MT+* and *MT−* alleles of *MADS2* and were used to determine that the major 18 bp indel in *MADS2* was fixed between the two mating-types in 14 independent isolates (Figure S2). It seems likely that polymorphisms in *MADS2* contribute to cis-regulatory differences that restrict its expression to *MT−*, but this idea remains to be directly tested.

Finally, two additional shared R-domain genes, *MT0796* and *MT0828*, were found to have strong zygotic expression with little or no cDNA detected in vegetative and gametic samples (Figures 4D, S5D, and Table S5). Neither predicted protein has a recognizable domain or homology outside of *Chlamydomonas*.

In summary, we have uncovered several potential new examples of sexual cooption for mating locus genes in *Chlamydomonas* that acquired sex-regulated expression patterns.

### Population genetics of the *MT* region

Genes in non-recombining sex-determining regions or sex chromosomes of haploid organisms are unsheltered and not expected to undergo loss or degeneration at the same rate that they do on Y and Z chromosomes [33]. Nonetheless, they are still subject to the effects of linkage disequilibrium that reduces the efficiency of natural selection in non-recombining regions (reviewed in [34], [35]) and are also expected undergo genetic differentiation between haplotypes [26], [33]. In a previous study we compared haplotype divergence in the *MT* locus of *Chlamydomonas reinhardtii* with its syntenic counterpart in *Volvox carteri* [11]. That study revealed a large discrepancy in divergence rates between shared genes in *Chlamydomonas MT* whose rates were low, versus those in *Volvox MT* whose rates were high, despite the two genomic regions sharing a common origin [14], [21]. The comparatively low rate of haplotype divergence in *Chlamydomonas MT* might be explained by rare genetic exchanges that cannot be detected in laboratory crosses but which act to reduce inter-haplotype diversity at the mating locus, an effect similar to that which has been observed in “ever-young” tree frog sex chromosomes [36].

#### Nucleotide diversity in *MT* and autosomal genes

In order to detect possible evidence of rare genetic exchange in *Chlamydomonas MT* we investigated patterns of nucleotide diversity in natural isolates. For this analysis we sequenced all or part of seven genes in thirteen wild isolates–seven *MT+* and six *MT−* strains collected from diverse geographic regions (Table S6). We also made use of published data from an additional four genes [37]. Population data were compiled for eleven genes total, including one *MT−* limited gene (*MID*), one *MT+* limited gene (*MTA1*), two randomly-selected shared genes in the R-domain (*PR46*, *PDK1*), three genes in the C or T domains (*SAD1*, *SPP3*, *MAT3*), and four autosomal genes that are unlinked to *MT* (*GP1*, *IDA5*, *CBLP*, *YPT4*). The mating locus genes that were used in this analysis are highlighted in Figure 1. We used these data to ask whether genes in *Chlamydomonas MT* show patterns of genetic diversity that are indicative of low recombination rates and selective sweeps, and to provide information about genetic exchange that might take place between the two *MT* haplotypes.

Nucleotide diversity (π) is a function of natural selection, mutation rates, recombination rates and population size/structure [38]. Diversity at synonymous coding sites and non-coding sequences (silent diversity or π_sil_) is considered neutral or nearly neutral and can be used to assess population structure. Theoretical and empirical data support the expectation of lower nucleotide diversity in non-recombining regions due to selective sweeps, background selection, decreased effective population size and Muller's ratchet effects [34], [39].

π_sil_ (multiplied by 1000 in Table 1) varied about fifty-fold across the genes examined here with values ranging from ∼1 to ∼50, but was lowest for *MT+* alleles of R-domain genes *PR46* (1.13) and *PDK1* (1.27) (Tables 1 and S7). π_sil_ for *PR46* and *PDK1* in *MT−* samples (6.08 and 18.8 respectively) was significantly higher than for the *MT+* samples, though still lower than π_sil_ for autosomal genes. The low π_sil_ values for *PR46* and *PDK1* suggest a recent selective sweep of the *MT+* haplotype. π_sil_ values for the sex-limited genes *MID* (11.1) and *MTA1* (4.22) were also relatively low and attributable to possible selective sweeps and/or lower effective copy number compared with shared *MT* genes and autosomal genes. Despite being in a nominally non-recombining region *SAD1* and *SPP3* had π_sil_ values of ∼30 to 50 that are not distinguishable from those of autosomal genes (*GP1*, *IDA5*, *CBLP*, *YPT4*). Moreover, the *SAD1* and *SPP3* π_sil_ values did not show differences between *MT+* and *MT−* when grouped by mating-type as we saw for *PR46* and *PDK1* (Tables 1 and S7). These data indicate that *SPP3* and *SAD1* are relatively uncoupled from the effects of presumed selective sweeps in the *MT* locus. The three *MT−* isolates of the C domain gene *MAT3* had relatively low diversity (5.80) compared with the *MT+ MAT3* isolates (24.6) and compared with the other two C/T domain genes *SPP3* and *SAD1*. The low diversity of *MAT3* from *MT−* isolates could be due to a selective sweep, but the nearby gene *SAD1* is closer to the R-domain than *MAT3* and has a π_sil_ value of ∼50 indicating that the low π_sil_ value for *MT−* isolates of *MAT3* is not associated with the *MT* region as a whole. Because π_sil_ for *MAT3* was based on only three isolates [37], we recalculated π_sil_ for the same three *MT−* isolates of *SAD1* in order to control for sampling bias. However, sub-sampling of *SAD1* from the isolates as used for *MAT3* increased rather than decreased its π_sil_ value (67.3, standard deviation 9.0) allowing us to rule out sample bias as the cause of low nucleotide diversity in *MT−* isolates of *MAT3*. Additional data will be required to resolve whether the low diversity we see for *MAT3* from *MT−* isolates is due to other causes such as a highly localized selective sweep in this gene.

**Table 1:** Population genetic data for *MT* and autosomal genes.

	no. sequences^1^		π sil^2^			F_ST_ 3
	*MT*+	*MT*−	total	*MT*+	*MT*−	
**R Domain sex-limited**						
*MTA1*	7	na	na	4.22 (1.11)	na	na
*MID*	na	6	na	na	11.11 (3.38)	na
**R Domain shared**						
*PR46*	7	6	16.0 (1.80)	**1.13** (0.24)	**6.08** (1.94)	**0.85000**
*PDK1*	7	6	23.3 (3.42)	**1.27** (0.32)	18.8 (4.51)	**0.72130**
**C/T domain shared**						
*SPP3*	7	6	45.6 (4.20)	44.7 (7.03)	51.8 (7.36)	−0.10563
*MAT3*	4	3	23.7 (3.43)	24.6 (5.88)	**5.80** (2.32)	**0.45217**
*SAD1*	7	6	43.8 (6.96)	31.9 (8.72)	50.1 (16.2)	0.16461
**Autosomal**						
*GP1*	7	6	25.1 (5.25)	31.3 (7.82)	20.8 (4.44)	−0.11345
*IDA5/Actin*	4	3	35.6 (5.38)	38.2 (7.15)	37.4 (15.0)	−0.11494
*CBLP*	4	3	49.9 (6.32)	49.7 (14.27)	49.0 (16.0)	0.01946
*YPT4*	4	3	22.7 (4.50)	16.9 (4.86)	26.2 (11.6)	0.12821

Notes: na not applicable.

1 Number of *MT+* and *MT−* sequences analyzed for each gene.

2 Polymorphism rate for silent sites (non-coding and synonymous)×1000. Standard deviation in parentheses. Values are given for all sequences (total) and for the *MT+* and *MT−* isolates separately. *MT*+ and *MT*− values that differ from the total value by >1 standard deviation are shown in bold.

3 Population differentiation between *MT+* and *MT−* isolates.

Values near 0 correspond to no differentiation and values near 1 correspond to complete differentiation. Bold values correspond to those genes showing significant differentiation between *MT+* and *MT−* isolates.

We calculated two indices of gene flow and population structure, d_A_ and F_ST_, to determine the extent to which genetic exchange between *MT+* and *MT−* isolates is constrained [38], [40], [41]. While sequence diversity in autosomal genes (*IDA5*, *CBLP* and *YPT4*, *GP1*) was independent of mating-type as indicated by d_A_ and F_ST_ values near zero (Tables 1 and S7), the R-domain genes *PR46* and *PDK1* showed strong *MT*-associated differentiation as evidenced by F_ST_ values that are between 0.5 and 1.0 (Table 1) and by d_A_ values that differ significantly from the null value of 0 (Table S7). These findings indicate that genetic exchange between shared R-domain genes is limited compared with autosomal genes that assort freely between *MT+* and *MT−* haplotypes (Tables 1 and S7). Consistent with our findings on nucleotide diversity the C/T domain genes *SAD1* and *SPP3* showed no evidence of mating-type-linked differentiation, while the C domain gene *MAT3* gene showed an intermediate level of mating-type-linked differentiation (F_ST_ = 0.45) (Tables 1 and S7).

**Figure 5:**
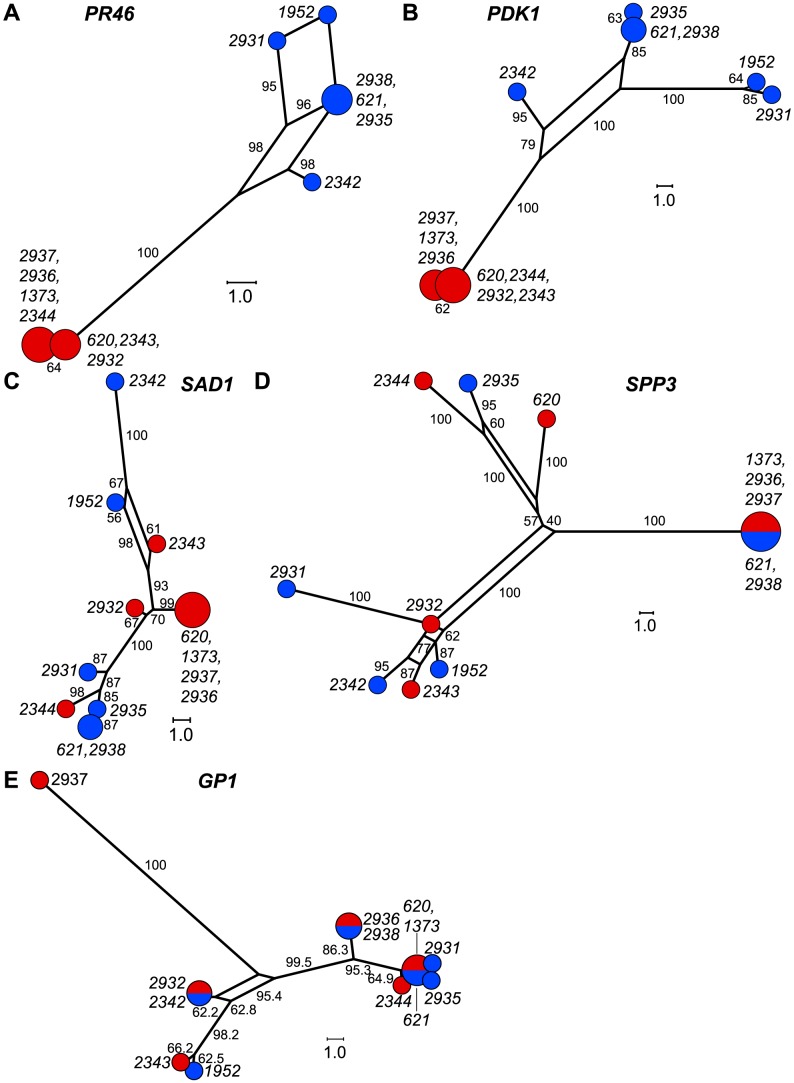
Haplotype networks of *MT* and autosomal genes. A–E. Unrooted parsimony splits networks of R-domain genes A. *PR46* and B. *PDK1*, C/T domain genes C. *SAD1* and D. *SPP3*, and autosomal gene E. *GP1*. Distances between nodes represent number of nucleotide changes. Bootstrap values from 1000 replicates are shown next to edges and expressed as rounded percentages. Circular nodes represent individual isolates with red and blue shading to indicate *MT+* and *MT−* respectively. Node size is proportional to the number of isolates in the node.

We graphically depicted the genetic relationships between *MT+* and *MT−* allelic diversity by constructing unrooted parsimony-based networks that are similar to phylogenetic trees, but accommodate incongruities by incorporating alternative paths or splits [42]. As suggested above, the networks for the R-domain genes *PR46* and *PDK1* show clear differentiation between *MT+* and *MT−* isolates (Figure 5A,B) with tight clustering of the *MT+* alleles. In contrast, genes in the T- and C-domains (*SAD1* and *SPP3*) show complete intermixing between *MT+* and *MT−* isolates (Figure 5C,D), with no apparent association of specific polymorphisms with mating-type. The *MAT3* gene did show some *MT*-associated differentiation, but this differentiation did not extend to the nearby *SAD1* gene (Figure S3) or to the *SPP3* gene indicating that these three loci are all separable from each other and from the R-domain by recombination. As expected none of the polymorphisms present in autosomal genes (*GP1*, *YPT4*, *IDA5*, *CBLP*) showed association with mating haplotype (Figures 5E and S3).

#### Gene conversion in the R-domain

The preceding data revealed far more genetic exchange in the C and T domains of *MT* than would be expected based on laboratory tests of recombination. However, these data do not explain why R-domain genes such as *PR46* and *PDK1* show orders of magnitude lower amounts of sequence differentiation compared with R-domain genes in *Volvox MT* [11]. Crossovers in the R-domain are likely to be lethal or highly deleterious due to rearrangements and deletions, but a second means of genetic exchange is gene conversion, where tracts of sequence from one allele can be unidirectionally transferred to a homologous partner in the diploid phase of the sexual cycle–most likely during meiosis. Such exchanges are expected to be infrequent, but could still help maintain sequence homogeneity between allelic gene pairs in the R-domain.

**Figure 6:**
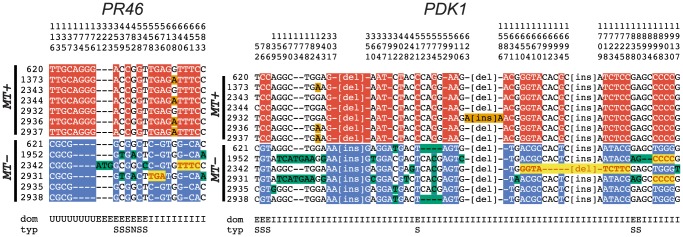
R-domain gene conversion between *MT*+ and *MT−* haplotypes. Polymorphic positions in alignments of R-domain genes *PR46* and *PDK1* from 7 *MT+* and 6 *MT−* isolates described in Table S7. The position in the alignment is displayed vertically above each column reading downward. The domain (dom) of the gene in which the polymorphism occurs is indicated below each column as follows: E (exon), I (intron) and U (untranslated region). For exonic positions the type of substitution (typ) is indicated as synonymous (S) or non-synonymous (N). Small insertion/deletion polymorphisms are indicated by dashes, while larger insertion/deletion polymorphisms are abbreviated as [ins] or [del]. Red background shading indicates polymorphisms specific to *MT+* isolates and blue background shading indicates polymorphisms specific to *MT−* isolates. Bold red sequences with yellow background shading show gene tracts where *MT−* sequences converted to *MT+*. Orange and green shading show polymorphisms segregating within *MT+* and *MT−* subgroups.

Gene conversion can be identified by comparing polymorphisms that are nearly fixed between the two mating-types and then identifying tracts where two or more adjacent polymorphisms have switched their pattern from one haplotype to the other [43]. Here we identified four short regions of gene conversion in the two R-domain genes that were randomly selected for this study–two tracts in *PDK1* and two tracts in *PR46* (Figures 6 and S6). None of the tracts were in repeat regions or microsatellites (Figure S6), and in all four cases the direction of conversion was from *MT+* to *MT−*. One of the gene conversion tracts in *PDK1* is present in both CC1952 and CC2931 (Figure 6) making its occurrence likely to predate the split between these two isolates. These previously undocumented gene conversion events may have important implications for mating locus evolution that are further elaborated below.

### Relationship between sequence rearrangements and suppressed recombination in *MT*

Blocked recombination in sex determining regions is believed to be maintained so that genes in these regions with sex-specific functions can remain tightly linked [4], [44]. Sequence rearrangements in heteromorphic sex chromosomes and in heteromorphic mating loci such as *Chlamydomonas MT* could accumulate passively as the result of blocked recombination, or they could be the primary cause of blocked recombination [26], [45]. In the latter case normal recombination should be restored in matings with isomorphic *MT* haplotypes while in the former case restoring collinearity at *MT* would not relieve suppression of recombination.

Mating between parents with the same *MT* haplotype in *Chlamydomonas* provide a means to test whether *MT* sequences are capable of normal recombination when their meiotic partner is collinear and homologous. Prior work established the basis of mating-type specification in *Chlamydomonas* and allowed the engineering of strains in which each parent contributes the same *MT* haplotype in a cross [19], [46]. *MT+* strains carrying a *Mid* transgene (*MT+::Mid-T*) were used as pseudo-*minus* parents in *MT+::Mid-T*×*MT+* crosses. *MT− mid-1 Fus-T* strains were used as pseudo-*plus* parents in *MT− mid-1 Fus-T*×*MT−* crosses (see Materials and Methods). The auxotrophic markers *nic7* and *thi10* (nicotinamide and thiamine requiring, respectively) flank the mating locus [47] and were used to identify potential crossovers within *MT* (Figure 1, Table S8). Recombination data for *MT+×MT+* and *MT−×MT−* crosses are summarized in Figure 1 and Tables S8 and S9.

To confirm the absence of recombination across *MT* in control strains, we crossed the *MT+* and *MT−* strains CC-123 *thi10 NIC7 MT+* and CC-2663 *THI10 nic7 MT−*. Out of 1040 random progeny, none were Nic− Thi−, while ten were Nic+ Thi+ and mated as *minus* strains. Of those ten, nine were diploid or aneuploid based on the presence of both the *nic7* and *NIC7* alleles. This leaves at most one true recombinant (0.1% frequency), a value that is consistent with previous data [48].

#### *MT+*× *MT+* crosses

We performed an *MT+*×*MT+* cross (*nic7 THI10 MT+::Mid-T*×*NIC7 thi10 MT+*), and scored 352 random progeny for nicotinamide and thiamine auxotrophy that would be indicative of recombination in or around *MT*. Thirteen Nic+ Thi+ and three Nic− Thi− putative recombinants were examined further. The *NIC7* locus was amplified and scored from the thirteen Nic+ Thi+ strains, two of which were found to contain both parental alleles meaning that they were either diploids or aneuploids. Excluding these two progeny we found 14 recombinants (11 Nic+ Thi+, 3 Nic−Thi−) out of 350 corresponding to a recombination frequency of ∼4% across *MT+* and a genetic distance close to the genome-wide average of ∼100 kb per cM [49].

Because the two *MT+* strains used above were isogenic, the sites of crossovers could not be determined. Therefore, a second cross was performed using an inter-fertile *MT+* wild isolate, CC-2344, as the *plus* parent and a recombinant *nic7 thi10 MT+ Mid-T* progeny from the first *MT+*×*MT+* cross as the *minus* parent. A total of 17 out of 377 random progeny were recombinant: 7 were Nic+ Thi−, and 10 were Nic− Thi+ giving a recombination rate of ∼4.5% that was similar to what we observed in the first cross. The recombinant progeny were further analyzed by scoring several additional polymorphic markers in *MT* (Figure 1, Table S8). These markers defined a minimum of four different breakpoint intervals, three of which lie entirely within the R-domain of the *MT+* haplotype (Figure 1, Table S8). One additional *MT*-linked marker, *MAT3*, and three autosomal markers—*YPT4*, *GP1* and *MMP1*—were scored to confirm normal meiotic segregation in this cross (Table S8). In summary, these data establish that meiotic recombination is possible for the *MT+* haplotype and that it is normally suppressed in *MT+*×*MT−* crosses.

#### MT−× MT− crosses

A similar experiment as above was done using *MT−* strains *nic7 MT−* and *NIC7 MT− mid1 Fus-T* as parents. The *thi10* marker was not available in this cross, so we instead used the *mid1* pseudo-*plus* mating phenotype as a second *MT*-linked marker to score recombination (Figure 1). Recombinants in this cross would be Nic+ progeny that mate as *minus*, or Nic− progeny that mate as *plus*. 600 progeny from a total of 206 zygotes were scored for mating phenotype and for nicotinamide auxotrophy. 599 of the progeny had the parental markers. A single putative recombinant progeny that was Nic+ and mated as a *minus* strain (*NIC7 MT−*) was found to contain both parental *NIC7* alleles and is presumed to be a diploid. Therefore, no meiotic recombinants were found between *MID* and *NIC7* in crosses with homologous *MT−* mating haplotypes (Table S9). The ∼240 kb region of *MT−* covered by these two markers includes ∼80 kb of collinear sequence flanking *MT−* (T domain) and ∼160 kb of R-domain sequence. The absence of recombination in this cross is incompatible with an average physical/genetic distance ratio of 100 kb/cM (Chi squared = 14.75, p value = 0.000122). Moreover, this segment of *MT−* was repressed for recombination at least as much as two previously described autosomal markers that show the largest known physical/genetic distance ratio in *Chlamydomonas* of 511 kb/cM [49] (Chi squared = 2.81, p value = 0.093).

The absence of recombination between collinear *MT−* partners could be caused by sequences in *MT−* that repress recombination in cis, but could also have been caused by the absence of *MT+* genes that promote recombination in trans (though no candidates for such genes are known). Both cis and trans effects on recombination have been reported previously in the non-recombining mating type chromosome of *Neurospora tetrasperma* [50]. To distinguish cis versus trans effects on recombination in *MT−*×*MT−* crosses we repeated the above cross with the *minus* parent CC1952 that has well-characterized molecular markers for mapping [49], [51] and the pseudo-*plus* strain *NIC7 MT− mid1 Fus-T*. We first scored a chromosome VI marker, *4121*, that was reported to be 27 cM from *MT* in conventional crosses [49]. 26/96 progeny from the *MT−*×*MT−* cross were recombinant for *4121* and *MT* resulting in a genetic distance of 27 cM. This result is consistent with normal recombination on Chromosome VI outside the mating locus (Table S9). A pair of autosomal markers on Chromosome III, *GAR1* and *GSAT*, also had a normal recombination distance of ∼20 cM (Table S9). However, the *MT* markers *MAT3* and *PDK1* had no recombinants (0/146)(Figure 1 and Table S9).

Taken together our data show that the *MT−* locus is a region of suppressed recombination that inhibits meiotic crossovers even when homologous collinear sequences are available for pairing. In contrast, the *MT+* locus shows normal meiotic recombination when it has a collinear pairing partner. This asymmetry between *MT+* and *MT−* may have consequences for other aspects of *MT* sequence evolution and differentiation that are elaborated in the Discussion.

## Discussion

### *MT* and its genetic content redefined

Key findings for our analysis of *MT* structure were identification of two new autosomal insertions in the *MT+* haplotype, *MTP0428* and the *SRL* region, that redefine the borders of *MT* with ∼30 additional kb of R-domain sequence in the *MT+* haplotype. Altogether, the *MT+* R-domain is approximately twice the size of the *MT−* R-domain due to three major autosomal translocations and the 16 kb repeat region (Figure 1). This degree of size asymmetry in a mating locus of a unicellular organism is atypical and has been reported to our knowledge in only one other instance for the smut fungus *Microbotryum* [9]. On the other hand, X and Y chromosomes of different sizes in haploid bryophytes are well-documented [12], but very little is known about how such size differences evolve in haploid systems. One prediction of Bull's theory of haploid dioecy is that non-recombining haploid X-Y chromosomes would expand by sequence additions rather than deletions and degeneration [33]. Our findings here support the role of sequence insertions causing *MT+* expansion, as does previous work on *Volvox MT* whose increased size relative to *Chlamydomonas MT* is largely due to accumulation of repeats and transposons with little evidence of gene loss [11]. However, Bull's theory predicts similar overall fates for haploid sex determining chromosomes and does not explain the emergence of size asymmetry that is evident, for example, in around half of the surveyed X-Y chromosome pairs from bryophytes [12]. The size and structural asymmetry of *Chlamydomonas MT* haplotypes could represent a model for how such size asymmetry evolves. In the last section we speculate on the basis for emergent asymmetry in the *Chlamydomonas* mating locus.

### New mating locus genes with potential functions in the sexual cycle

The *SRL* region of *MT+* is of special interest as it was created from a partial fragment of an autosomal gene, *SRR16*, which then underwent further fragmentation into three sub-regions. *SRLb* represents an intriguing example where gene fragmentation, a process typically associated with decay, may lead to the creation of new genes in an environment such as *MT* where recombination is greatly reduced and where neutral or even slightly deleterious mutations have a greater chance of achieving fixation in the population compared with autosomal regions [35].

In *Chlamydomonas MT* controls sexual differentiation, fertilization competence and uniparental organellar DNA inheritance [18]. Genes whose presence or expression is limited to only one mating-type are candidates for governing these aspects of the sexual cycle, and in this study we identified several candidates.

Interestingly, within each of the translocations and the 16 kb repeat region of *MT+* are candidates. For example *MTP0428*, *MTA4* and *EZY2* are zygotically expressed, while *SRLb*, *MTA1* and *OTU2* are up-regulated in gametes and zygotes (Figures 1, 4ABDE, S4A, S5A, and Table S5).

We found that two *Chlamydomonas*-specific genes encoding proteins of unknown function, *MT0796* and *MT0828* are both expressed zygotically (Figure 4, Table S5) in a pattern similar to the early zygotic genes *EZY1* and *EZY2* that are speculated to have a role in uniparental chloroplast DNA inheritance [19], [52]. MT0796 and/or MT0828 may also be involved in this process or in other early zygote functions that include zygote wall formation, flagellar resorption, karyogamy and chloroplast fusion [18].

Expression of the putative MADS-box transcription factor-encoding gene *MADS2* was restricted to *MT−* cells and zygotes, and not detectable in *MT+* cells (Figures 4C, S5F, and Table S5). The function of MADS2 in *MT−* cells is unknown, but the potential connection to green algal sexual cycles is intriguing given the major role for MADS box proteins in plant reproductive development [53]. A second shared gene of interest is *OTU2* that encodes a putative otubain-related deubiquitylating protease [18], [54]. The *OTU2* mRNA in *MT+* gametes is expressed at levels several fold higher than that in *MT−* gametes (Figure S4A), possibly as a result its higher copy number in *MT+* cells. This biased expression pattern is consistent with a role for OTU2 in mating-type differentiation or the sexual cycle.

Among the sex-regulated shared genes in *MT*, only two have *Volvox* homologs–*MADS2* and *HRGP1*–and these *Volvox* homologs are either in or adjacent to the mating locus [11]. *MADS2* in *Volvox* shows female-biased expression, which is opposite to the pattern in *Chlamydomonas* (where *MT+* is homologous to *Volvox* female *MT* and *MT−* is homologous to *Volvox* male *MT*). It is possible that MADS2 controls a sex-related process such as uniparental mitochondrial DNA inheritance where the inheritance pattern has switched from the *MT−* parent in *Chlamydomonas* [18] to the female parent in *Volvox* [55]. *HRGP1* encodes a putative cell wall protein that is up-regulated in gametes of both mating types of *Chlamydomonas* [11], [19], but which shows male-biased, gametic expression in *Volvox* [11]. This change from equal expression in both gametes to male-biased expression suggests that *HRGP1* participates in *Volvox* gametogenesis but may be required in higher amounts for spermatogenesis than oogenesis.

### Gene conversion and genetic exchange in *Chlamydomonas MT*

**Figure 7:**
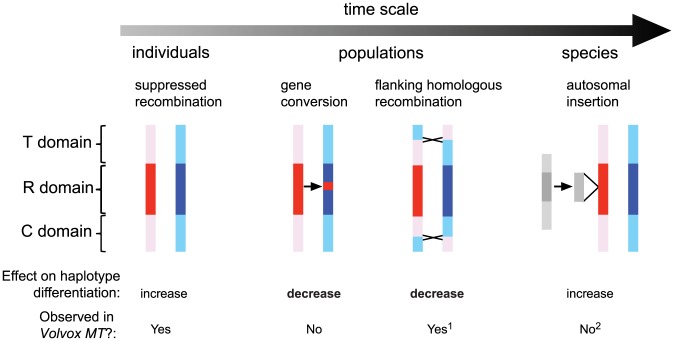
Genetic processes that shaped evolution of the *Chlamydomonas reinhardtii* mating locus. The time scale arrow on top represents a frequency continuum for genetic processes affecting *MT* that are detectable within individual generations, within populations, or in the species. Models of genetic exchange show the *MT* haplotypes in red (*MT+*) and blue (*MT*−) with the rearranged (R) domain shaded dark and the flanking telomere-proximal (T) and centromere-proximal (C) domains shaded light. From left to right: In individuals little or no genetic exchange is observed in crosses due to suppressed recombination; In populations occasional gene conversion within the R-domain, and crossover exchange or gene conversion in the T and C domains act to homogenize genetic variation that accumulates between haplotypes; At the species-level autosomal insertions (gray shaded regions) have occurred at least three separate times in the *MT*+ haplotype and spread to fixation, thereby adding new mating-type-limited genes to the locus. The lower section summarizes the impact of genetic interactions in *Chlamydomonas MT* in terms of increasing or decreasing haplotype differentiation and whether such interactions occur in *Volvox MT*. Notes: 1, Suppressed recombination in *Volvox MT* does not appear to extend beyond the R-domain as it does in *Chlamydomonas* [11]. 2, Only unique autosomal sequence insertions (but not transposons or repeats) are considered in this schematic.

The expectation for genes in non-recombining regions such as *MT* is allelic differentiation into two haplotypes [26]. Our population data confirm this expectation for shared genes in the R-domain that show overall clustering by mating type (Tables 1 and S7, Figure 5). However, we uncovered evidence of gene conversion between *MT+* and *MT−* alleles of R-domain genes indicating that there is genetic exchange in the rearranged portion of *MT* that can act as a homogenizing force to counteract the effects of reduced recombination (Figures 5 and 7). We also found evidence for genetic exchange between C and T domain genes that almost never show recombination in laboratory crosses. The observed genetic exchanges in the C/T domains could be from crossovers or from gene conversion. In either case the amount of exchange in the C/T domains is significantly higher than in the R domain and is enough to partially or completely remove linkage between C- or T-domain polymorphisms and mating type (Figure 5, Tables 1 and S7). An important consequence of exchange between *MT+* and *MT−* polymorphisms in the C/T domains is that genes such as *SAD1* whose expression and function is limited to one mating type (*MT−* in the case of *SAD1*) remain under selection in both mating types, and this explains why the *MT+* locus retains a functional copy of *SAD1* [56], [57]. Moreover, the data presented here for the first time distinguish the recombination behavior of C and T domain genes that are largely uncoupled from mating type with those in the R-domain that show mating-type associated differentiation (Figure 5, Tables 1 and S7).

The data we obtained on gene conversion in *Chlamydomonas MT* parallels that found recently for the fungi *Cryptococcus neoformans* that has a relatively large heteromorphic mating locus [58], and for the non-recombining *mat* locus of *Neurospora tetrasperma* [59]. Moreover, infrequent gene conversion between heteromorphic or rearranged regions may be a more general property of sex chromosomes as it has been seen in animal sex chromosomes [60], [61] where has been proposed to act as a means of genetic homogenization [36].

### Resolution of the mating locus age paradox in Volvocine algae

Our data documenting genetic exchange in *Chlamydomonas MT* help resolve a paradox regarding the degree of differentiation between mating haplotypes in the two Volvocine algal species *Chlamydomonas reinhardtii* and *Volvox carteri* [14], [21]. We propose that gene conversion in *Chlamydomonas MT* acts to promote sequence homogeneity between shared genes and thus maintains a “youthful” appearance for such genes despite their time of residence in the *MT* locus. In contrast, no such mechanism appears to have operated during the recent history of the *V. carteri* lineage where differentiation of *MT* genes is orders of magnitude higher and extends back through speciation events [11].

Why do the *Chlamydomonas* and *Volvox MT* regions differ in their behavior with respect to genetic exchange? Although their structural organizations are similar, *Volvox MT* is about five times larger than *Chlamydomonas MT*, has a much higher repeat content, and retains very little residual synteny or gene order between rearranged genes compared with *Chlamydomonas* [11]. We speculate that a combination of reduced effective population size and of selection on mating locus genes for oogamous traits in *Volvox* promoted *MT* expansion past a critical size/structural threshold where residual exchange between shared genes by gene conversion could no longer occur as it does in *Chlamydomonas*. Once past such a threshold the differentiation rates between mating haplotypes would be expected to accelerate and further reduce the potential for gene conversion or recombination. Determining the structure of *MT* in other Volvocine algae with different colony organization and reproductive morphologies may shed light on the parameters that caused *MT* to evolve so differently between *Chlamydomonas* and *Volvox*, and help determine when the recombination dynamics of *MT* began to diverge in the lineage.

### Mechanisms of recombination suppression and emergent asymmetry in *MT*

While rearrangements in the *MT* locus may contribute to suppressed recombination, we found evidence here for at least one other mechanism that suppresses recombination in the *MT−* haplotype even when it has a collinear partner. We propose that one or more sequences within *MT−* are responsible for suppressing recombination and may have originally evolved to maintain linkage between the *MT−* sex determining genes *MID* and *MTD* [18], [62], similar to what has been proposed to occur during the early evolution of diploid sex chromosomes [4], [45]. Subsequent rearrangements that generated the R-domain could have arisen passively as a result of blocked recombination, or arisen under selection to strengthen linkage between genes in each *MT* haplotype.

We speculate that *MT−* mediated recombination suppression (as opposed to rearrangements) is responsible for the extremely low observed recombination rates in the collinear C/T domains of *Chlamydomonas MT* that flank the R domain. In contrast to the *Chlamydomonas* C/T domains, recombination in sequences immediately adjacent to *Volvox MT* is not suppressed [11]. We predict that this difference in recombination behavior for collinear sequences flanking *MT* in the two species is that *Volvox MT* lacks sequences that intrinsically repress recombination. If so, recombination would occur normally in *Volvox MT* for either *MTF*×*MTF* or *MTM*×*MTM* crosses if such matings could be arranged. Testing this idea will be a goal for future studies.

While Y or W chromosome degeneration is the prevalent mechanism behind heteromorphic sex chromosomes in diploid systems [3], no comparable mechanism explains how heteromorphic sex chromosomes might evolve in a haploid system such as primitive plants [33]. The unique sequence properties of the *MT−* haplotype that suppress homologous recombination may have generated other asymmetries found in the *MT* locus. It is striking that of the three independent autosomal insertion events in *MT* and the 16 kb repeat expansion, all occurred in *MT+* that we have demonstrated retains competence for initiation of meiotic recombination. Additionally, all the gene conversion events that we have documented are asymmetric with respect to direction of sequence transfer from *MT+* to *MT−*. While these observations showing asymmetrical behavior of *MT+* and *MT−* haplotypes are limited, they fit a pattern that might be explained in terms of differential access of their sequences to meiotic recombination and DNA repair machinery that could bias the location of non-homologous insertions and gene conversion events.

Interestingly, there are hints of similar types of asymmetry as we have documented for *Chlamydomonas MT* in mating type chromosomes from other species. In the fungus *Microbotryum* there is size asymmetry between the two mating type chromosomes that are estimated to be ∼3.3 and ∼4.0 Mb respectively, though detailed sequence information about the two haplotypes is still lacking [63]. Mating locus chromosomes in the heterokaryotic self-fertile fungus *Neurospora tetrasperma* are blocked for recombination and have rearrangements between the *mat a* and *mat A* haplotypes that help ensure linkage between the *mat* locus and the centromere so that meiotic progeny remain heterokaryotic [7], [50]. Differences in the amount of repeat accumulation in the *mat a* and *mat A* chromosomes and in codon usage for genes from the two haplotypes have been reported [7], [31], but the reasons for this intriguing asymmetry are unclear.

Our data indicate that asymmetry in both size and recombination behavior can arise in the evolution of haploid mating systems and perhaps influence the preferential expansion of one mating haplotype over the other. Whether the mechanisms that cause mating locus size asymmetry in *Chlamydomonas* contribute to the formation of heteromorphic chromosomes in haploid systems such as primitive plants or fungi remains to be determined.

## Materials and Methods

### *Chlamydomonas* strains

Strains used for the population studies are listed in Table S6 and were obtained from the *Chlamydomonas* Stock Center (http://chlamycollection.org/strains/). Strains used to test recombination in the mating locus are as follows: CC-123, *thi10 MT+*; CC-2663, *nic7 MT−*. Note that the *ac29* mutation present in the original CC-2663 strain reverted [64]; B32, *mid-1 MT−* with a *FUS1* transgene [46]. B32 mates as a *plus* strain; PF1, *nic7 MT+* with a *MID* transgene. PF1 was created with a *MID* transgene (3.5 kb ApaI fragment from plasmid pmid7.1 [46]) that was cotransformed into CC-1865 (*arg2 fus1-1 MT+*) along with pArg7.8 that contains a wild-type argininosuccinate lyase gene [65]. A *MID*-expressing Arg^+^ transformant was crossed to CC-85 (*nic7 MT+*) to create PF1; K33, *nic7 MT+ thi10* with a *MID* transgene. K33 was a progeny from a cross of CC-123 to PF1 that mates as *minus*, deposited with the *Chlamydomonas* Stock Center as CC-3947.

### Mating and genetic analysis

*Chlamydomonas* strains were grown on TAP plates supplemented as appropriate with nicotinamide (nic, 4 µg/ml), thiamine (thi, 5 µg/ml), and/or acetylpyridine (AcPy, 15 µl/l) to enhance scoring of the nic- phenotype. Crosses were done by standard procedures [66] and random progeny were scored for auxotrophies by growth on appropriate media, or for polymorphisms using PCR amplification (Table S10). Progeny exhibiting recombinant phenotypes were subcloned and retested to confirm their genotypes.

### Mating locus sequences and annotation

Sequences and annotation of the *plus* and *minus* mating locus haplotypes are described in [11] and available in Genbank under accession numbers GU814014 and GU814015. Gene models were further refined using predictions available from Phytozome [23] and EST support, and were confirmed where possible using data derived from 454 transcriptome data available at http://genomes.mcdb.ucla.edu/Cre454/project.html and deposited in the NCBI Short Read Archive (http://www.ncbi.nlm.nih.gov/sra) under accession SRA020135.

### Analysis of autosomal duplications

*Plus* and *minus* mating locus sequences were aligned to the V4 genome assembly from Phytozome [23] using BLAST in order to identify duplicated regions. Dot plots were generated using the dotmatcher program in the EMBOSS package [67] with default parameters. Putative coding regions were aligned using MUSCLE [68] and then manually verified and adjusted to correct placement of splice junctions. MEGA5 [69] was used to calculate divergence values for the alignments in Table S2 and Figure 3 using the Tamura 3-parameter model to estimate distances. dN and dS values were calculated using yn00 in the PAML package [70], [71]. CAI values were calculated using the CAICal webserver as described in [72].

### RNA preparation

*C. reinhardtii* cultures of CC620 (*MT+*) and CC621 (*MT−*) were grown to confluence on TAP plates [66] for one week under continuous light. Cells were washed off of the plates with nitrogen-free (N-free) HSM and placed immediately into either +N (for vegetative samples) or −N (for gametes and zygotes) HSM media [66] at ∼1.0×10^7^ cells/mL at 24°C for 3 hours in large unshaken Erlenmeyer flasks filled to ∼1/4 volume. After resuspension and incubation as described above, vegetative and gametic samples were collected from each culture. To generate zygotes, equal volumes of *plus* and *minus* gametes were briefly mixed in an Erlenmeyer flask and samples collected after 10′, 30′ 60′ and 120′. Mating progression was monitored from fixed samples at each time point and had reached ∼90% by 10′ (data not shown). For each sample, 100 mL of cells were collected in 2×50 mL polypropylene conical tubes and Tween-20 was added to a final concentration of 0.005%. The samples were centrifuged at 4,000× g for 3 minutes, the supernatant decanted, and the pellet snap frozen in liquid nitrogen. RNA was extracted with Trizol (Invitrogen, Carlsbad CA) according to the manufacturer's protocol. RNA was further purified using RNAEasy columns (Qiagen) according to the manufacturer's protocol.

### cDNA synthesis

DNAseI (Roche) treated total RNA was reverse transcribed using Superscript III (Invitrogen, Carlsbad CA), with the following modifications to the manufacturer's protocol. A 9∶1 mixture of anchored dT_20_ (TTTTTTTTTTTTTTTTTTTTV) and random hexamer oligos were used to prime first strand cDNA synthesis. cDNA synthesis reactions were incubated as follows: 25C 10′, 42C 10′, 50C 15′, 55C 15′, 60C 15′, 65C 15′, 85C° 5′. RNAse H was subsequently added and reactions incubated 30′ at 37C. cDNAs were diluted 1∶10 in TE (10 mM Tris pH 8.0/1 mM EDTA) and stored at −20C prior to use.

### Quantitative RT-PCR (qPCR)

Table S10 lists all primers used. cDNAs were diluted 1∶10 in sterile filtered ddH_2_O and 10 µL was used for each of the 20 µL qPCR reactions. The reactions were performed in triplicate on each of two biological replicates. Reaction conditions were as described previously [73] and reactions were amplified using a Bio-Rad iCycler iQ Real Time Thermal Cycler w/Optical Module (BioRad, Hercules CA) using the following cycling conditions: 95C 10″, 60C 10″, 72C 30″ for 40 cycles. Melt curves and gel electrophoresis were used to confirm the presence of a single amplification product of the correct size in each reaction. For all primer sets a standard dilution curve was prepared using cDNAs pooled from all samples. Relative cDNA levels were calculated using the best-fit curve from the standard dilution of each primer set and then normalized against the 18S cDNA signal.

### Genomic DNA isolation and PCR amplification from *C. reinhardtii* isolates

Genomic DNA was isolated by CsCl banding [74]. Table S10 lists all primers used for amplification of target genes. PCR products from two independent reactions per sample were sequenced to confirm that no errors were introduced into the sequence during amplification.

### Population genetic data and phylogenetic networks

Sequence alignments were done using ClustalX [75] and manually adjusted. DnaSP [76] was used to calculate values in Tables 1 and S7. π_sil_ data were calculated from alignment files with gaps and non-synonymous sites removed. d_XY_, d_A_ and F_ST_ were calculated from full alignments with gaps removed. Three gene conversion tracts were identified by DnaSP using the algorithm of Betran [43]. The fourth tract was present in 2 out of 6 *MT−* isolates and was identified manually. The manually identified tract meets Betran's criteria for gene conversion since four consecutive occurrences of a polymorphism are present in 1/3 of the *MT−* isolates with a p-value of .012 (0.33^4^ = 0.012) [43]. Sequences used were derived from this study and from a previous study [37] with strains and accession numbers in Table S6. Phylogenetic networks were constructed using the program SplitsTree [42]. The ParsimonySplits approach was used to calculate the network from ungapped alignments with 1000 bootstrap replicates, and the networks were rendered using the Equal Angle and Convex Hull methods. Network topology was unchanged when calculated using distance-based approaches such as the Neighbor-net method (data not shown).

## Supporting Information

Figure S1 The *Chlamydomonas reinhardtii* life cycle. The upper panel (shaded pale blue) shows the vegetative reproductive cycle where cells of either mating type grow and undergo multiple fission (one or more alternating rounds of DNA replication and mitotic division) to produce 2^n^ daughter cells. Four daughters are depicted here, but the number varies depending on growth conditions. The lower panel (shaded pale yellow) shows the sexual cycle where nitrogen depletion (−N) induces gametic differentiation. Gametes of opposite mating type recognize each other through flagellar adhesive proteins called agglutinins and fuse to form a quadriflagellate zygote that differentiates into a dormant diploid zygospore (shaded orange). Upon return to light and nutrients the zygospore undergoes germination and meiosis to produce 2 *MT+* and 2 *MT−* haploid cells that hatch and reenter the vegetative reproductive cycle.
(EPS)

Figure S2 MADS2 polymorphisms. A. Alignment of *MADS2* 5′ region from *MT+* and *MT−* sequences beginning with the transcription start site. The predicted start codon is bold and intronic sequences are lower case. Polymorphic positions are counter-shaded black. Binding sites for PCR primers used to assess the major indel polymorphism between *MT+* and *MT−* isolates are indicated by forward and reverse arrows. B. PCR amplification products, strain names, and mating type are indicated in the lower panel that shows presence/absence of the indel in *MT+* and *MT−* isolates.
(PDF)

Figure S3 Polymorphic sites from genes used in this study. Polymorphic sites for the indicated genes from natural isolates are displayed as described in the legend for Figure 6, but without color or shading. Alignments are shown for *SAD1* (C-domain gene), *SPP3* (T-domain gene), *MID* (R-domain gene, *MT−* limited), *MTA1* (R-domain gene, *MT+* limited), *GP1* (autosomal gene), and Mito (mitochondrial sequence). The segment of *SAD1* chosen for sequencing is within the agglutinin head domain and does not contain repetitive shaft domain sequences [56]. In the *SPP3* alignment, the numbers shown after position 535 indicate how many TG dinucleotide pairs follow base 533 in the labeled strain.
(PDF)

Figure S4 Quantitative and semiquantitative RT-PCR data for *OTU2a* and *MTA4*. Samples are labeled as in Figure 4. A. *OTU2* expression determined using primers that amplify both the *MT+* and *MT−* copy of the gene. B. 18S rRNA internal control. Error bars are the standard error of the mean for the technical triplicates. C and D. Semiquantitative RT-PCR data for *MTA4* and 18S rRNA with different amplification cycle numbers shown on the left. Samples are the same as in Figure 4.
(PDF)

Figure S5 Quantitative RT-PCR for biological replicates. qRT-PCR results for biological replicates. Panels A–F show expression values from quantitative RT-PCR (qRT-PCR) experiments for indicated genes calculated as described in Materials and Methods. Each panel groups genes by their overall expression pattern as follows: A, *MT+* gametic; B, *MT−* gametic; C, *MT−* only; D, early zygotic; E, zygotic; F, reduced in zygotes. RNA samples were derived from *MT+* vegetative cells (PV) and gametes (PG), *MT−* vegetative cells (MV) and gametes (MG), and from zygotes at 10 minutes, 30 minutes, 1 hour, 2 hours and 3 hours after mating (Z10, Z30, Z1h, Z2h and Z3h respectively). * No expression detected.
(PDF)

Figure S6 Full alignments of *PR46* and *PDK1* showing gene conversion tracts. Full alignments of R-domain genes *PR46* and *PDK1* from 7 *MT+* and 6 *MT−* isolates described in Table S6 and Figure 6. Insertion/deletion polymorphisms are indicated by dashes. Red background shading indicates polymorphisms specific to *MT+* isolates and blue background shading indicates polymorphisms specific to *MT−* isolates. Yellow background shading shows gene tracts where *MT−* sequences converted to *MT+*. Orange and green shading show polymorphisms segregating within *MT+* and *MT−* subgroups respectively. Tan shading highlights a single *PDK1* polymorphism that segregates in both *MT+* and *MT−* isolates. * symbol is below non-polymorphic positions.
(PDF)

Table S1 Locations and protein IDs of autosomal genes and their *MT+* duplicates. The JGI v4 *C. reinhardtii* genome is the basis for the gene coordinates. PID: Protein Identification Number from the V4 genome assembly models. ps: pseudogene.
(PDF)

Table S2 Divergence between autosomal genes and their *MT+* duplicates. Alignments of cDNAs and genomic DNAs were used to define the intergenic and intronic DNA sequences. CDS: coding sequence. Intron: non-coding sequence between the start and stop codons of the CDS. Intergenic: Non-coding sequence outside of the CDS. Divergence scores determined as in [77]. Codon substitution rates were determined as in [71]. SE is the Standard Error. ND: Not determined.
(PDF)

Table S3 Codon Adaptive Indices (CAI) for autosomal genes and their *MT+* duplicates. A: Autosome, M: Mating Type Locus, ps: pseudogene.
(PDF)

Table S4 Annotations for *C. reinhardtii* mating locus genes. Sequences and annotation of the *MT+* and *MT−* locus haplotypes are described in [11], [19] and available in Genbank under accession numbers GU814014 and GU814015. + Augustus v5 Model IDs begin with “5”. * *MT−* coordinates are based upon the Genbank entry noted above. The Augustus v10.2 Model IDs were determined using the Algal Functional Annotation Tool at the following URL: http://pathways.mcdb.ucla.edu/chlamy/id_conversion.html. The start and stop codon locations of *MT+* gene models are based on the v4 JGI genome assembly. NA not applicable. ND Not determined.
(PDF)

Table S5 Summary of expression data for mating locus genes. JGI EST: Number of ESTs mapped to the gene model on the Phytozome browser. + one or more EST matches. − no EST matches. Probes from previous study [19] were matched to their overlapping gene model(s) in the JGI V4 *C. reinhardtii* genome assembly. #, Probe 65 was in the intergenic region between LEU1S and 522872 and most likely detected RNA from a transposable element. Expression stage is abbreviated as Veg, vegetative; Gam, gametic; Zyg, zygotic; all stages, All; ND, not detected; NA, not available 454: Number of 454 cDNA sequences that map to the gene model on the UCLA MCDB/MBI Genome Browser http://genomes.mcdb.ucla.edu/Cre454/project.html. + one or more 454 matches. − no 454 matches. All 454 sequences corresponding to duplicated *MT+* genes in the *SRL* and *MTA* regions were realigned to the *MT+* and autosomal gene copies, and polymorphisms were used to distinguish the origin of the transcript. Positive evidence of a transcript is indicated only when genomic origin could be determined. JGI v4 PID: JGI *C. reinhardtii* v4 Protein ID (if available) for the listed gene model. ^a^ from [19].
(PDF)

Table S6 *Chlamydomonas reinhardtii* strains and DNA sequences used for population genetic studies. Chlamydomonas Resource Center (http://chlamycollection.org/) strain numbers are listed along with common laboratory names for selected strains. Geographic origins are abbreviated as follows: FL, Florida; MA, Massachusetts; MN, Minnesota; NC, North Carolina; PA, Pennsylvania; QC, Quebec, Canada. Genbank accession numbers are listed for genes from each isolate. ^a^ Data from [37].
(PDF)

Table S7 Population data and haplotype differentiation for mating locus and autosomal genes. na not applicable. 1. Number of *MT+* and *MT−* strains analyzed for each gene. 2. Total number of silent sites (non-coding and synonymous) 3. Number of segregating silent sites. 4. Polymorphism rate for silent sites. Standard deviation in parentheses. 5. Tajima's D statistic calculated for silent substitutions. Significant value (p<.05) is in bold. nd indicates not done for groups with less than 4 sequences. 6. d_xy_ Average pairwise substitution rate between *MT+* and *MT−* isolates with Jukes-Cantor correction. 7. d_A_ residual difference between *MT+* and *MT−* isolates when corrected for within-population divergence. Standard deviation in parentheses. Bold values are samples with dA scores outside of one standard deviation from the null value of zero. 8. Population differentiation between *MT+* and *MT−* isolates.
(PDF)

Table S8 Recombination data for *MT+* homozygous cross. Parental strains K33 and CC-2344 (both *MT+*), were crossed and progeny that showed recombination between *NIC7* and *THI10* were scored for additional markers in the indicated genes. The first 8 markers are in *MT* and listed in the order they occur on chromosome 6. *MMP1*, *YPT4* and *GP1* are unlinked to *MT* and were used as controls to show independent assortment of autosomal markers in the cross. Nic and Thi columns indicate auxotrophy (−) or prototrophy (+) for nicotinamide and thiamine respectively.
(PDF)

Table S9 Recombination data for *MT−* homozygous cross. 1. recombinant progeny/total progeny. 2. Expected recombinants for *MAT3-PDK1* and for *MID-NIC7* are based on the genome-wide average of ∼1 cM/100 kb. For *4121-MT* and *GAR1-GSAT* the expected value is based on previous data [49].
(PDF)

Table S10 List of oligonucleotides used in this study. ^a^ primers derived from [78]. ^b^ primers derived from [79]. ^c^ primers derived from [73].
(PDF)
