# Supplementary material for: Species and Population Level Molecular Profiling Reveals Cryptic Recombination and Emergent Asymmetry in the Dimorphic Mating Locus of C. reinhardtii
Source: PLoS Genet. 2013 Aug 29;9(8):e1003724. doi: 10.1371/journal.pgen.1003724 (PMC3757049; doi:10.1371/journal.pgen.1003724)
Supplement: Figure S2 — MADS2 polymorphisms. A. Alignment of MADS2 5′ region from MT+ and MT− sequences beginning with the transcription start site. The predicted start codon is bold and intronic sequences are lower case. Polymorphic positions are counter-shaded black. Binding sites for PCR primers used to assess the major indel polymorphism between MT+ and MT− isolates are indicated by forward and reverse arrows. B. PCR amplification products, strain names, and mating type are indicated in the lower panel that shows presence/absence of the indel in MT+ and MT− isolates. (PDF) [file pgen.1003724.s002.pdf]

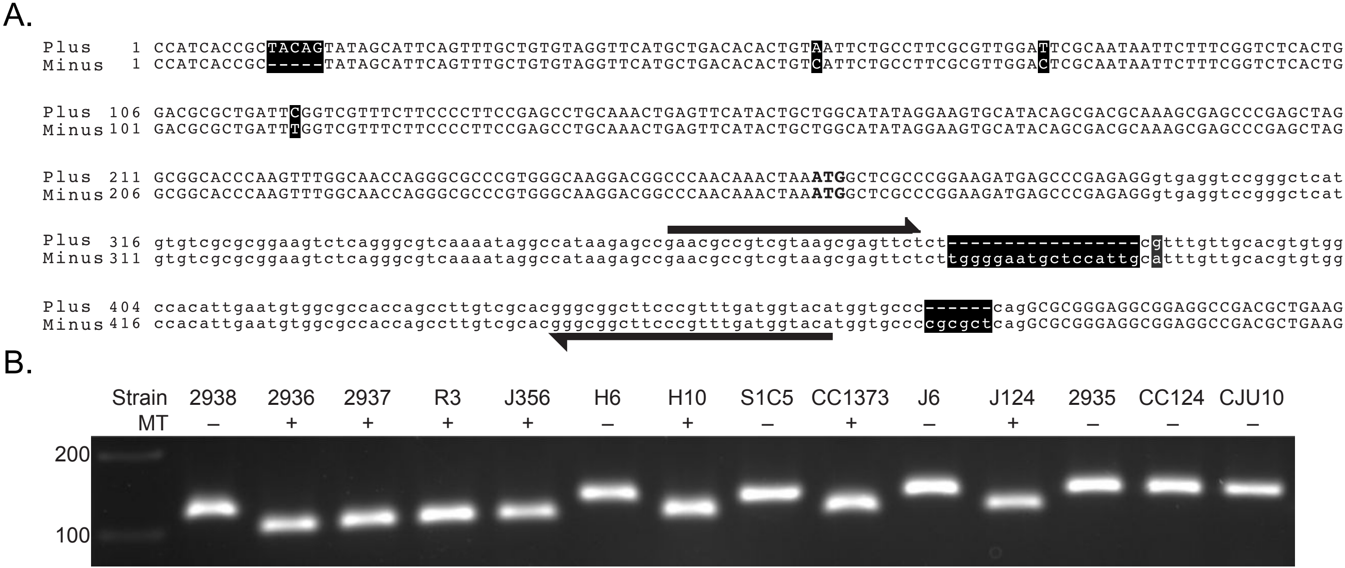
A.
Plus 1 CCATCACCGCTACAGTATAGCATTCAGTTTGCTGTGTAGGTTCATGCTGACACACTGTAATTCTGCCTTCGCGTTGGATTCGCAATAATTCTTTCGGTCTCACTG
Minus 1 CCATCACCGC-----TATAGCATTCAGTTTGCTGTGTAGGTTCATGCTGACACACTGTCATTCTGCCTTCGCGTTGGACTCGCAATAATTCTTTCGGTCTCACTG
Plus 106 GACGCGCTGATTCGGTCGTTTCTTCCCCTTCCGAGCCTGCAAACTGAGTTCATACTGCTGGCATATAGGAAGTGCATACAGCGACGCAAAGCGAGCCCGAGCTAG
Minus 101 GACGCGCTGATTTGGTCGTTTCTTCCCCTTCCGAGCCTGCAAACTGAGTTCATACTGCTGGCATATAGGAAGTGCATACAGCGACGCAAAGCGAGCCCGAGCTAG
Plus 211 GCGGCACCCAAGTTTGGCAACCAGGGCGCCCGTGGGCAAGGACGGCCCAACAAACTAAATGGCTCGCCCGGAAGATGAGCCCGAGAGGgtgaggtccgggctcat
Minus 206 GCGGCACCCAAGTTTGGCAACCAGGGCGCCCGTGGGCAAGGACGGCCCAACAAACTAAATGGCTCGCCCGGAAGATGAGCCCGAGAGGgtgaggtccgggctcat
Plus 316 gtgtcgcgcggaagtctcagggcgtcaaaataggccataagagccgaacgccgtcgtaagcgagttctct-----------------cgtttgttgcacgtgtgg
Minus 311 gtgtcgcgcggaagtctcagggcgtcaaaataggccataagagccgaacgccgtcgtaagcgagttctcttggggaatgctccattgcatttgttgcacgtgtgg
Plus 404 ccacattgaatgtggcgccaccagccttgtcgcacgggcggcttcccgtttgatggtacatggtgccc------cagGCGCGGAGGCGGAGGCCGACGCTGAAG
Minus 416 ccacattgaatgtggcgccaccagccttgtcgcacgggcggcttcccgtttgatggtacatggtgccccgcgctcagGCGCGGAGGCGGAGGCCGACGCTGAAG
B.
Strain 2938 2936 2937 R3 J356 H6 H10 S1C5 CC1373 J6 J124 2935 CC124 CJU10
MT – + + + + – + – + – + – – –
200
100
