# Supplementary material for: Species and Population Level Molecular Profiling Reveals Cryptic Recombination and Emergent Asymmetry in the Dimorphic Mating Locus of C. reinhardtii
Source: PLoS Genet. 2013 Aug 29;9(8):e1003724. doi: 10.1371/journal.pgen.1003724 (PMC3757049; doi:10.1371/journal.pgen.1003724)
Supplement: Figure S3 — Polymorphic sites from genes used in this study. Polymorphic sites for the indicated genes from natural isolates are displayed as described in the legend for Figure 6, but without color or shading. Alignments are shown for SAD1 (C-domain gene), SPP3 (T-domain gene), MID (R-domain gene, MT− limited), MTA1 (R-domain gene, MT+ limited), GP1 (autosomal gene), and Mito (mitochondrial sequence). The segment of SAD1 chosen for sequencing is within the agglutinin head domain and does not contain repetitive shaft domain sequences [56]. In the SPP3 alignment, the numbers shown after position 535 indicate how many TG dinucleotide pairs follow base 533 in the labeled strain. (PDF) [file pgen.1003724.s003.pdf]

SAD1

```
                                                                                          111
                    11111222222222333334444444444444444444444555555666666667778888888899990010
bp          136777783889900004689902679111111222333333345889912245800256669236111447990456153
          67839456787388901236785767929456789012013567805096935812237753499928136369370443004

MT+   620 CGCGG---GGGGCAGCTGCGTAGCCGCGC---GGATGA--T----TGCTAGCGCCCGCATACCCTCCGCGGCACGGCCGCCAT
     1373 CGCGG---GGGGCAGCTGCGTAGCCGCGC---GGATGA--T----TGCTAGCGCCCGCATACCCTCCGCGGCACGGCCGCCAT
     2343 CGCGGCAGGACACAGCTGCGGCGCTCCGC---GGATGA--T----TACTAGTTTTGACATACCCTCCGTAGTGCGGCCGTCAT
     2344 CGCAG---GTCAC------AGAGTCGCGC---GGATGA--T----TGCTAGCGCCCATGTGGTCTTTATGATACGGCGTCAGT
     2932 CGCAG---CTCAC------GGAGTCGCGC---GGATGA--T----TGCTAGCGCCCACATACCTCCCGTGGTGCGACCGTCGT
     2936 CGCGG---GGGGCAGCTGCGTAGCCGCGC---GGATGA--T----TGCTAGCGCCCGCATACCCTCCGCGGCACGGCCGCCAT
     2937 CGCGG---GGGGCAGCTGCGTAGCCGCGC---GGATGA--T----TGCTAGCGCCCGCATACCCTCCGCGGCACGGCCGCCAT
MT–   621 ATCAG---GTCAC------GGAGTCGCGC---GGATGA--T----TGCTAGCGCCCATGTGGTCTTTGTGGCACGGTGTCCGG
     1952 CGCGG---GTCAC------GGAGTCGCGCCATGGATGAGAGCATCCGTCGTTTTTGACAAACCCTCCGCGGCACGGCCGCCAT
     2342 CGTGA---GACAGAGCTGCGGCACTCAAT---------GAGCATCCGTCGTTTTTGGCAAACCTCCCGTGGTGCGACCGTCAT
     2931 CGCAG---GTCAC------GGAGTCCCGC---GGATGA--T----TACTAGCGCCCATGTGGTCTTTGTGGTAGAGCCGCCGT
     2935 CGCAG---GTCAC------GGAGTCGCGC---GGATGA--T----TGCTAGCGCCCATGTGGTCTTTGTGGCACGGTGTCCGG
     2938 ATCAG---GTCAC------GGAGTCGCGC---GGATGA--T----TGCTAGCGCCCATGTGGTCTTTGTGGCACGGTGTCCGG

dom       EEEEEEEEEEEEEEEEEEEEEEEEEEEEEEEEEEEEEEEEEEEEEEEEEEEEEEEEEEEEEEEEEEEEEEEEEEEEEEEEEEE
typ       NNSSS   NNNSN      SSSNSSSSS                NNNSSNNSNSSSSSNSNSNSSSSSNNSSSSNSSNSSNSS
```

MID

```
          1111112335566
          8888880294836
          1234676060082

MT–   621 ----TTACACGGT
     1952 ----TTGCACAGC
     2342 CGCGAAAGTTGGC
     2931 ----TTGCACATC
     2935 ----TTACACGGT
     2938 ----TTACACGGT

dom       IIIIIIIIEEIUU
typ               SS
```

GP1

```
             11111111111111111111222222222223334444444445566666777
bp         3611223334466688888899012344444673792233334690701268678
          39869671394513823467868947315789945740723451590256506723

MT+   620 CCCGG-TAGTTGAGAACGTGTAGAGC-CT---ACCGGGT----CG--TTGGACTCA
     1373 CCCGG-TAGTTGAGAACGTGTAGAGC-CT---ACCGGGT----CG--TTGGACTCA
     2343 TGTGGGCAGTTGAGTACGTGCTCAAC-CT---GCTGGGCGATGTA--TCACACCGA
     2344 CCCGG-TAGTTGAGTACGTGTAGAGC-GT---ACCGGGT----TG--TTGGACTCA
     2932 CCTGGGCACTTGAATACGTGCTCAAC-CT---GCCGGGCGATGTG--TTACACCGA
     2936 CCCGG-TAGTTAAGTACGTGTTCAAC-CT---ACCAGGT----TG--TTGGACTCA
     2937 CCCAAGCCGGCGTAGGCCGCATCTACACCATTGTCGTAC----TGGTCTAGGTCCA
MT–   621 CCCGG-TAGTTGAGAACGTGTAGAGC-CT---ACCGGGT----CG--TTGGACTCA
     1952 CGTGGGCAGTTGAGTATGTGCTCAAC-CT---GCTGGGCGATGTA--TCACACCGA
     2342 CCTGGGCACTTGAATACGTGCTCAAC-CT---GCCGGGCGATGTG--TTACACCGA
     2931 CCCGG-TAGTTGAGTACGTGTAGAGC-CT---ACCGGGT----CG--TTGGACTCG
     2935 CCCGG-TAGTTGAGTACGTGTAGAGT-CT---ACCGGGT----CG--TTGGACTCA
     2938 CCCGG-TAGTTAAGTACGTGTTCAAC-CT---ACCAGGT----TG--TTGGACTCA

dom       EEEEEIIIIIIIIIIIIIIIIIIIIIIIIIIIIEIIIIIIIIIIIIEEEEEEEEE
typ       SSNNN                             S            SSNNNSSNN
```

Mito

```
          3566677777899
bp        2846956669103
          2649176783829

MT+   620 TAATAC---AAGG
     1373 TCAACAT--CCAG
     2343 TAAACAT--CCAG
     2344 TAAACAT--CCAG
     2932 TAAACATTTCCAG
     2936 TCAACAT--CCAG
     2937 AA-TCAT--CCGT
MT–   621 TAATAC---AAGG
     1952 AA-TCAT--CCGT
     2342 TAAACAT--CCAG
     2931 TAAACATTTCCAG
     2935 TCAACAT--CCAG
     2938 TAATAC---AAGG

dom       EEUUUUUUUUUUE
typ       SS          N
```

MTA1

```
          22234
          22366
          12504

MT+   620 AAGAC
     1373 ATGAC
     2343 AAGCC
     2344 GAACC
     2932 AAGCT
     2936 ATGAC
     2937 ATGAC

dom       IIIEE
typ          SN
```

SPP3

```
                                111111111111222222222222222222222222222333333333333333333333
bp            112222233333344449345799999999001112222222222333333577788011111223344478888888
          1234045678901238901232768023456789176890123456789012345304768034568351201731234567

MT+   620 GT--ATGCCCGG---T-----CGGGGGCAGCCTCCAACAGCAGCAGCAG---CAACTCGCGTTGCGAACTATGAACTGTGCC
     1373 AAC-AT---------T-----AGACG--------CGACAG---------------CTTGTGC-GCAGGCTTATTATGCCTGA
     2343 GTCCGGGCCCGGT-G------CGGCT--------CAACAGCAG------------CCCCCGTTAT-AGATAATTATGCC---
     2344 GTCCATGCCCGG---T-----CGGGG--------CAACAGCAGCAGCAG---CAACTCGCTTTGCGAACTAATAACTGTGCC
     2932 GTCCGGGCCCGG--GG-----CGGCG--------CAGCAGCAGCAGCAGCAGCAGCCCCCGT-GCGAACTAATTATGCC---
     2936 AAC-AT---------T-----AGACG--------CGACAG---------------CTTGTGC-GCAGGCTTATTATGCCTGA
     2937 AAC-AT---------T-----AGACG--------CGACAG---------------CTTGTGC-GCAGGCTTATTATGCCTGA
MT–   621 AAC-AT---------T-----AGACG--------CGACAG---------------CTTGTGC-GCAGGCTTATTATGCCTGA
     1952 GTCCGGGCCCGGGGGTGGGGGCGGAG--------CAACAGCAGCAGCAG------CCCCCGTTAT-AACGAATTATGCC---
     2342 GTCCGGGCCCGG--G------CGGCG--------TAA------------------ACCCCGTTAT-AGATAATTATGCC---
     2931 GTCCGGGCCCGG--G------AGGCG--------CAACAGCAG------------CCCCCTTTAT-AACTAATTATGCC---
     2935 GTCCATGCCCGG---T-----CTGGG--------CAACAGCAGCAGCAGCAGCAACTCGCGTTGCGAACTAATAGCTGTGCC
     2938 AAC-AT---------T-----AGACG--------CGACAG---------------CTTGTGC-GCAGGCTTATTATGCCTGA

dom       IIIIIIIIIIIIIIIIIIIIIEEEEEEEEEEEEEEEEEEEEEEEEEEEEEEEEEEIIIIIIIIIIIIIIIIIIIIIIIIIII
typ                            SSSSS        NNN                 S

          4444444445555555555555555-55666666666666666667777777777777777888888888888889999999999999
bp        0233699990122222222333333-99001111122233467880011111112222233011224666999990000000011111
          6526912366823456789012345-787912789579011420479034567801345692573964573456912345789012348

MT+   620 CTTCCATGGCGTGT-TG-----------CAGTTTAAGCAACTCCGGGTTTG-GTTC---GGTAACCTATTAA--TCCCCA-------AC
     1373 CCTCCACCCAG-----------------CGATTG-AGGAAATCCAGGTTTG-GTTCTCGCGTAACCTATTAA--TCCCCA-------AC
     2343 CCTCCATGGATTGTGTGTGTGTGTG4-ACAGGTGCAGCAACCCCGGGTTCG-GTTCTCGGCGAAGCTATTAAAAC---AACACCCTC-C
     2344 TCTTCGTGCCTTGTGTGTGTGTGTG32ACACGGGCTTGGGATGTGGGTTTG-GTTC---GGTAACTTATTAA--TCCCAA-------AC
     2932 CCTCCATGGATTGTGTGTGTGTGTG2-ACAGGTGCAGCAACCCCGGGT----ACGTTCGGGGAAGCTATTAAAAC---AACACCCTC-C
     2936 CCTCCACCCAG-----------------CGATTG-AGGAAATCCAGGTTTG-GTTCTCGCGTAACCTATTAA--TCCCCA-------AC
     2937 CCTCCACCCAG-----------------CGATTG-AGGAAATCCAGGTTTG-GTTCTCGCGTAACCTATTAA--TCCCCA-------AC
MT–   621 CCTCCACCCAG-----------------CGATTG-AGGAAATCCAGGTTTG-GTTCTCGCGTAACCTATTAA--TCCCCA-------AC
     1952 CCGCCATGGATTGTGTGTGTGTGTG10ACAGGTGCAGCAACCCCGGGTTTG-GTTCTCGGGGAAGCTATTAAAAC---AACACCCTC-C
     2342 CCTCGATGGATTGTGTGTGTGTGTG2-ACAGGTGCAGCAACCCCGATGGTGTACGTTCGGGGAAGCTATTAAAAC---AACACCCTC-C
     2931 CCTCCATGGATTGTGTGTGTGTGTG--ACAGGTGCAGCAACCCCGGGT----ACGTTCGGGGGGCCAGACCCCCCCCCACCACACACAT
     2935 CTTCCGTGCCGTGTGTGTGTGCG----ATACGGGCTGGGGATGCGGGTTTG-GTTC---GGTAACCTATTAA--TCCCCA-------AC
     2938 CCTCCACCCAG-----------------CGATTG-AGGAAATCCAGGTTTG-GTTCTCGCGTAACCTATTAA--TCCCCA-------AC

dom       IIIIEEEEEEIIIIIIIIIIIIIIIIIIIIIIIIIIIIIIIIIIIIIIIIIIIIIIIIIIIEEEEEEEIIIIIIIIIIIIIIIIIIIIIII
typ           SNNNNN                                                 SSSNNSS
```
