# Supplementary material for: Species and Population Level Molecular Profiling Reveals Cryptic Recombination and Emergent Asymmetry in the Dimorphic Mating Locus of C. reinhardtii
Source: PLoS Genet. 2013 Aug 29;9(8):e1003724. doi: 10.1371/journal.pgen.1003724 (PMC3757049; doi:10.1371/journal.pgen.1003724)
Supplement: Figure S4 — Quantitative and semiquantitative RT-PCR data for OTU2a and MTA4. Samples are labeled as in Figure 4. A. OTU2 expression determined using primers that amplify both the MT+ and MT− copy of the gene. B. 18S rRNA internal control. Error bars are the standard error of the mean for the technical triplicates. C and D. Semiquantitative RT-PCR data for MTA4 and 18S rRNA with different amplification cycle numbers shown on the left. Samples are the same as in Figure 4. (PDF) [file pgen.1003724.s004.pdf]

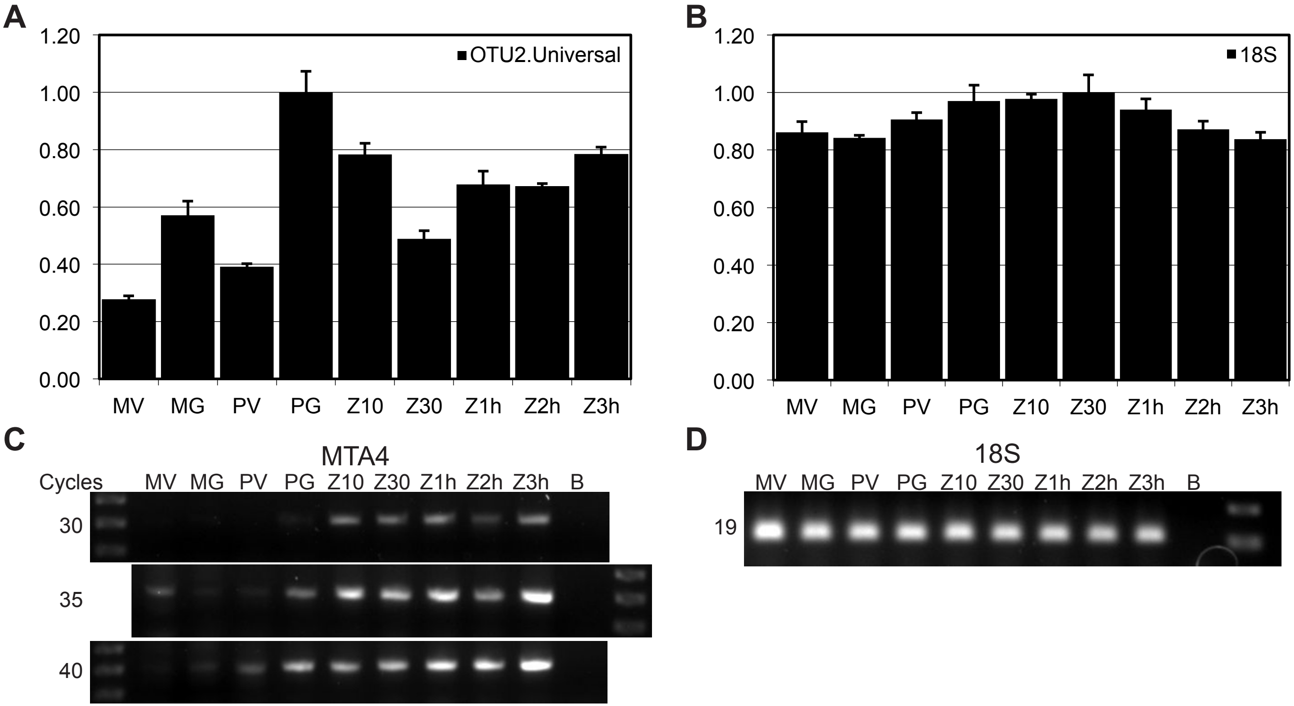
A
OTU2.Universal
1.20
1.00
0.80
0.60
0.40
0.20
0.00
MV MG PV PG Z10 Z30 Z1h Z2h Z3h
B
18S
1.20
1.00
0.80
0.60
0.40
0.20
0.00
MV MG PV PG Z10 Z30 Z1h Z2h Z3h
C
MTA4
Cycles MV MG PV PG Z10 Z30 Z1h Z2h Z3h B
30
35
40
D
18S
MV MG PV PG Z10 Z30 Z1h Z2h Z3h B
19
