# Supplementary figures and images for: Species and Population Level Molecular Profiling Reveals Cryptic Recombination and Emergent Asymmetry in the Dimorphic Mating Locus of C. reinhardtii
Source: PLoS Genet. 2013 Aug 29;9(8):e1003724. doi: 10.1371/journal.pgen.1003724 (PMC3757049; doi:10.1371/journal.pgen.1003724)

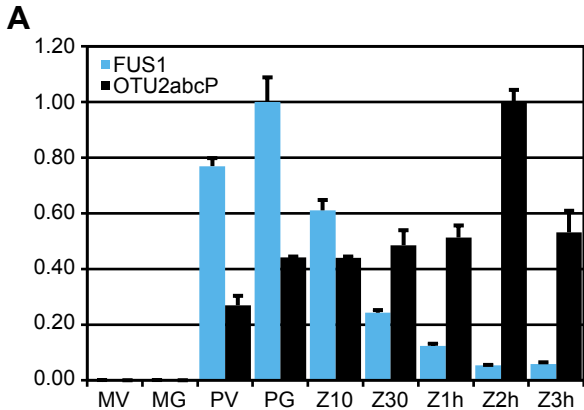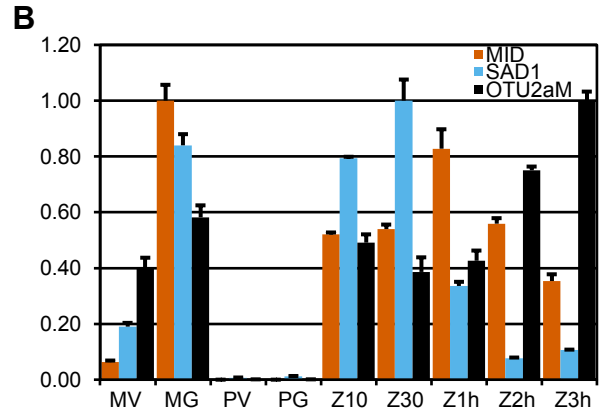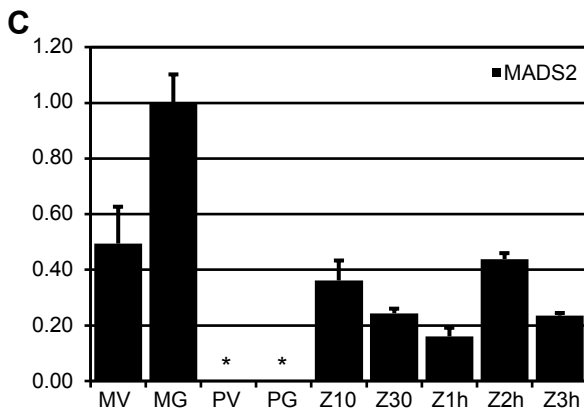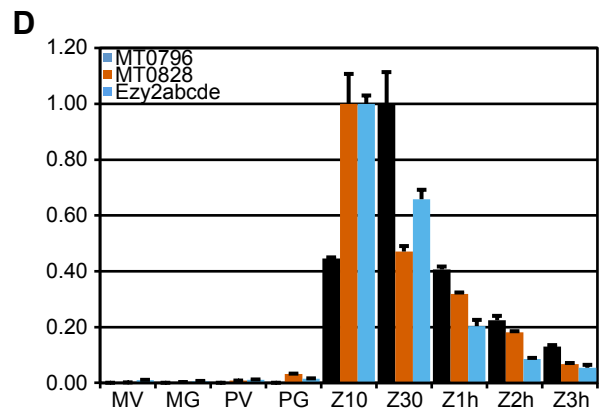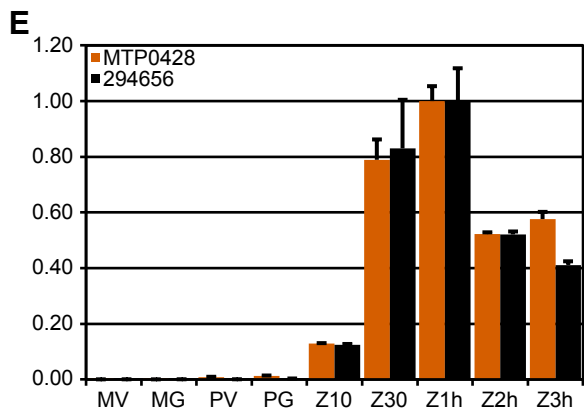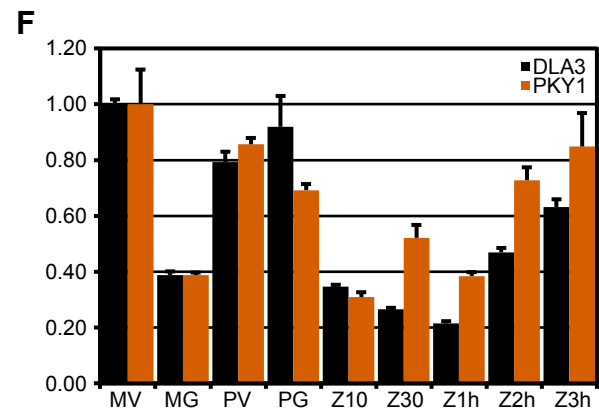

Supplement: Figure S5 — Quantitative RT-PCR for biological replicates. qRT-PCR results for biological replicates. Panels A–F show expression values from quantitative RT-PCR (qRT-PCR) experiments for indicated genes calculated as described in Materials and Methods. Each panel groups genes by their overall expression pattern as follows: A, MT+ gametic; B, MT− gametic; C, MT− only; D, early zygotic; E, zygotic; F, reduced in zygotes. RNA samples were derived from MT+ vegetative cells (PV) and gametes (PG), MT− vegetative cells (MV) and gametes (MG), and from zygotes at 10 minutes, 30 minutes, 1 hour, 2 hours and 3 hours after mating (Z10, Z30, Z1h, Z2h and Z3h respectively). * No expression detected. (PDF) [file pgen.1003724.s005.pdf]
