# Supplementary material for: Species and Population Level Molecular Profiling Reveals Cryptic Recombination and Emergent Asymmetry in the Dimorphic Mating Locus of C. reinhardtii
Source: PLoS Genet. 2013 Aug 29;9(8):e1003724. doi: 10.1371/journal.pgen.1003724 (PMC3757049; doi:10.1371/journal.pgen.1003724)
Supplement: Figure S6 — Full alignments of PR46 and PDK1 showing gene conversion tracts. Full alignments of R-domain genes PR46 and PDK1 from 7 MT+ and 6 MT− isolates described in Table S6 and Figure 6. Insertion/deletion polymorphisms are indicated by dashes. Red background shading indicates polymorphisms specific to MT+ isolates and blue background shading indicates polymorphisms specific to MT− isolates. Yellow background shading shows gene tracts where MT− sequences converted to MT+. Orange and green shading show polymorphisms segregating within MT+ and MT− subgroups respectively. Tan shading highlights a single PDK1 polymorphism that segregates in both MT+ and MT− isolates. * symbol is below non-polymorphic positions. (PDF) [file pgen.1003724.s006.pdf]

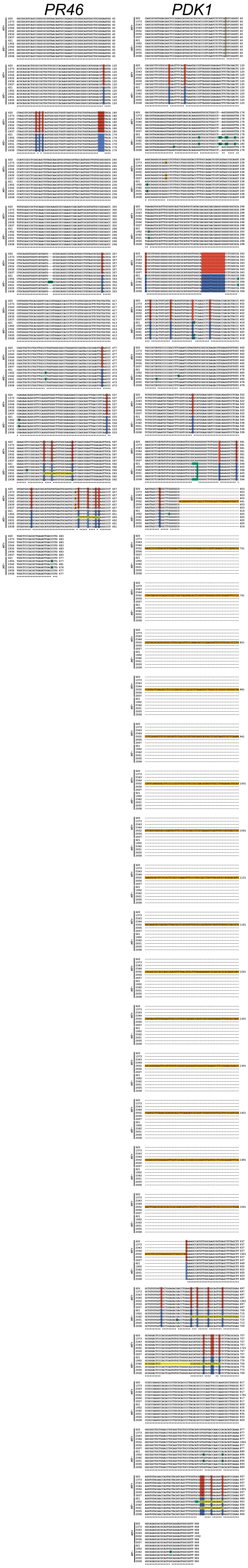
PR46
PDK1
