# Supplementary material for: Species and Population Level Molecular Profiling Reveals Cryptic Recombination and Emergent Asymmetry in the Dimorphic Mating Locus of C. reinhardtii
Source: PLoS Genet. 2013 Aug 29;9(8):e1003724. doi: 10.1371/journal.pgen.1003724 (PMC3757049; doi:10.1371/journal.pgen.1003724)
Supplement: Table S1 — Locations and protein IDs of autosomal genes and their MT+ duplicates. The JGI v4 C. reinhardtii genome is the basis for the gene coordinates. PID: Protein Identification Number from the V4 genome assembly models. ps: pseudogene. (PDF) [file pgen.1003724.s007.pdf]

TABLE S1
Locations and predicted protein IDs of duplicated *MT*+ genes and their autosomal counterparts.

***MT* Gene**			**Autosomal Gene**	
**Model Name**	**JGI v4 PID**	**Location**	**JGI v4 PID**	**Location**
MTP0428	167487	Chromosome_6:428272-432213	294656	Chromosome_6:202103-206447
MTP0428	167487	Chromosome_6:428272-432213	522812	Chromosome_6:87314-88538
MTP0428	167487	Chromosome_6:428272-432213	522811	Chromosome_6:92118-93458
MTA5	522877	Chromosome_6:546710-551888	396153	Chromosome_16:2929948-2945527
MTA4	7933	Chromosome_6:551994-560141	185335	Chromosome_16:2945860-2953729
psMTA2	305935	Chromosome_6:561462-562582	195673	Chromosome_16:2954391-2957164
MTA1	195674	Chromosome_6:562980-564042	ND	ND
MTA3	195822	Chromosome_6:567034-569191	185334	Chromosome_16:2958242-2960432
294708	294708	Chromosome_6:577663-578485	152340	Chromosome_16:2961105-2966438
SRLa	None	Chromosome_6:821696-824156	168182	Chromosome_10:5383680-5385785
SRLb	None	Chromosome_6:815243-821387	168182	Chromosome_10:5385782-5388233
SRLc	None	Chromosome_6:798573-799852	168182	Chromosome_10:5388224-5389295

The JGI v4 *C. reinhardtii* genome is the basis for the gene coordinates. PID: Protein Identification Number from the V4 genome assembly models. ps: pseudogene.
