# Supplementary material for: Species and Population Level Molecular Profiling Reveals Cryptic Recombination and Emergent Asymmetry in the Dimorphic Mating Locus of C. reinhardtii
Source: PLoS Genet. 2013 Aug 29;9(8):e1003724. doi: 10.1371/journal.pgen.1003724 (PMC3757049; doi:10.1371/journal.pgen.1003724)
Supplement: Table S2 — Divergence between autosomal genes and their MT+ duplicates. Alignments of cDNAs and genomic DNAs were used to define the intergenic and intronic DNA sequences. CDS: coding sequence. Intron: non-coding sequence between the start and stop codons of the CDS. Intergenic: Non-coding sequence outside of the CDS. Divergence scores determined as in [77]. Codon substitution rates were determined as in [71]. SE is the Standard Error. ND: Not determined. (PDF) [file pgen.1003724.s008.pdf]

TABLE S2

Divergence between duplicated *MT*+ genes and their autosomal counterparts

							Divergence		Codon Substitution Rate				
***MT*+ Gene**	**Autosomal Paralog**	**Region**	**Alignment Length**	**Gaps**	**Total Gap Length**	**Substitutions**	**Score**	**SE**	**dN**	**SE**	**dS**	**SE**	**dN/dS**
						**MTP0428 Region**							
		Intergenic	788	7	462	33	0.1100	0.0188					
MTP0428	294656	CDS	3213	17	99	150	0.0501	0.0045	0.0443	0.0045	0.065	0.0091	0.682
		Intron	703	8	43	33	0.0519	0.0084					
						**MTA Region**							
MTA5	396153	CDS	1494	1	1500	39	0.0268	0.0039	0.0123	0.0033	0.0766	0.016	0.161
		Intron	984	11	37	75	0.0844	0.0093					
		Intergenic	468	5	54	24	0.0605	0.0117					
MTA4	185335	CDS	2643	5	32	49	0.0191	0.0026	0.0058	0.0024	0.0991	0.0243	0.059
		Intron	5220	38	564	343	0.0780	0.0046					
		Intergenic	1724	7	174	58	0.0385	0.0054					
psMTA2	195673	CDS	1011	1	7	22	0.0223	0.0048	0.0186	0.0058	0.0302	0.0089	0.616
		Intron	169	1	8	5	0.0318	0.0149					
		Intergenic	3672	20	1923	102	0.0609	0.0061					
MTA3	185334	CDS	501	1	15	5	0.0104	0.0047	0.0055	0.0039	0.0267	0.0155	0.206
		Intron	358	4	41	14	0.0456	0.0116					
		Intergenic	9628	22	7834	93	0.0540	0.0059					
294708	152340	CDS	185	0	0	3	0.0165	0.0091	0.0131	0.0093	0	0	ND
		Intron	164	0	0	3	0.0186	0.0106					
						**SRL Region**							
SRLc	168182	CDS	720	1	6	22	0.0316	0.0068	0.0261	0.0073	0.0361	0.0139	0.723
		Intron	481	5	44	26	0.0625	0.0117					
SRLb	168182	CDS	1049	2	4	35	0.0344	0.0057	0.0167	0.0068	0.0438	0.0182	0.381
		Intron	1398	3	11	79	0.0596	0.0066					
SRLa	168182	CDS	1528	1	18	43	0.0292	0.0045	0.0173	0.0039	0.0588	0.0119	0.294
		Intron	327	4	8	27	0.0908	0.0185					

Alignments of cDNAs and genomic DNAs were used to define the intergenic and intronic DNA sequences. CDS: coding sequence. Intron: non-coding sequence between the start and stop codons of the CDS. Intergenic: Non-coding sequence outside of the CDS. Divergence scores determined as per[77]. Codon substitution rates were determined as per[71]. SE is the Standard Error. ND: Not determined
