# Supplementary material for: Species and Population Level Molecular Profiling Reveals Cryptic Recombination and Emergent Asymmetry in the Dimorphic Mating Locus of C. reinhardtii
Source: PLoS Genet. 2013 Aug 29;9(8):e1003724. doi: 10.1371/journal.pgen.1003724 (PMC3757049; doi:10.1371/journal.pgen.1003724)
Supplement: Table S3 — Codon Adaptive Indices (CAI) for autosomal genes and their MT+ duplicates. A: Autosome, M: Mating Type Locus, ps: pseudogene. (PDF) [file pgen.1003724.s009.pdf]

TABLE S3

Codon adaptive indices (CAI) of translocated *MT*+ genes and their autosomal paralogs

Name	Location	Length	CAI
152340	A	183	0.731
294708	M	183	0.731
185334	A	498	0.505
MTA3	M	483	0.489
195673	A	960	0.535
psMTA2	M	960	0.532
185335	A	1245	0.740
MTA4	M	1239	0.743
396153	A	1491	0.649
MTA5	M	1485	0.649
294656	A	3174	0.573
MTP0428	M	3147	0.549
SRR16a	A	1527	0.566
SRLa	M	1509	0.571
SRR16b	A	1044	0.518
SRLb	M	1047	0.557
SRR16c	A	717	0.575
SRLc	M	711	0.573

A: Autosome, M: Mating Type Locus, ps: pseudogene
