# Supplementary material for: Species and Population Level Molecular Profiling Reveals Cryptic Recombination and Emergent Asymmetry in the Dimorphic Mating Locus of C. reinhardtii
Source: PLoS Genet. 2013 Aug 29;9(8):e1003724. doi: 10.1371/journal.pgen.1003724 (PMC3757049; doi:10.1371/journal.pgen.1003724)
Supplement: Table S4 — Annotations for C. reinhardtii mating locus genes. Sequences and annotation of the MT+ and MT− locus haplotypes are described in [11], [19] and available in Genbank under accession numbers GU814014 and GU814015. + Augustus v5 Model IDs begin with “5”. * MT− coordinates are based upon the Genbank entry noted above. The Augustus v10.2 Model IDs were determined using the Algal Functional Annotation Tool at the following URL: http://pathways.mcdb.ucla.edu/chlamy/id_conversion.html. The start and stop codon locations of MT+ gene models are based on the v4 JGI genome assembly. NA not applicable. ND Not determined. (PDF) [file pgen.1003724.s010.pdf]

TABLE S4
Annotations for the *C. reinhardtii* mating locus genes

MT+ gene	*MT+* Start	*MT+* Stop	*MT+* Strand	*MT-* Gene	*MT-* Start*	*MT-* Stop*	*MT-* Strand	JGI *MT+* (V4) Protein ID or Augustus v5 ID+	JGI *MT+* (V4) Location	Augustus v10.2 Model ID	Domain	Annotation
**Shared Genes**												
NIC7	339575	346160	-	NIC7	39983	47119	-	522848	chromosome_6:339304-346672	Cre06.g251450.t1.2	T	Quinolinate synthetase A
SPS1	346944	350043	+	SPS1	47524	50742	+	206416	chromosome_6:346944-350043	Cre06.g251500.t1.1	T	Spermine synthase
SPP3	350573	353784	-	SPP1C	54194	58754	-	196107	chromosome_6:350536-355861	Cre06.g251550.t1.2	T	SppA, protease IV, signal peptide peptidase, serine endopeptidase of S49 family (ClpP clan)1
EIF5Bb	358313	361792	+	EIF5Bb	61516	65003	+	206391	chromosome_6:358313-362377	Cre06.g251600.t1.1	T	Eukaryotic translation initiation factor eIF5-like, eIF2 epsilon-like;
PTC1	362764	372516	-	PTC1	66675	74040	+	196076	chromosome_6:362753-373273	Cre06.g251650.t1.2	T	Putative low-affinity phosphate transporter, similar to yeast Pho87, Pho90 & Pho91 transporters involved in activation of the PHO pathway. Contains an N-terminal SPX domain and a C-terminal SLC13 permease domain.
182394	374956	375825	+	182394	78390	78788	-	182394	chromosome_6:374753-376959	Cre06.g251700.t1.1	T	Protein of Unknown Function
97782	379597	380211	+	97782	81015	81629	-	97782	chromosome_6:379597-380210	Cre06.g251750.t1.1	T	Protein of Unknown Function
RFC4	381303	384361	-	RFC4	83198	86315	-	195837	chromosome_6:381041-385068	Cre06.g251800.t1.1	T	Homologous to eukaryotic RFC4 (DNA replication factor C complex subunit 4); accessory protein of DNA polymerase
182392	385245	386007	+	182392	86973	87519	+	182392	chromosome_6:385179-386977	Cre06.g251850.t1.1	T	Protein of Unknown Function
ALB3	387192	389979	+	ALB3	88680	91451	+	187295	chromosome_6:387003-390870	Cre06.g251900.t1.1	T	One (of two) proteins related to the Arabidopsis ALBINO3 protein. This protein is the product of the AC29 gene [PMID: 8647391]. Alb3 is a chloroplast protein required for the integration of the light-harvesting complex protein into the thylakoid membrane [PMID: 12215522]
PSF2	392335	397177	-	PSF2	94067	98926	-	522858	chromosome_6:392283-397491	Cre06.g251950.t1.2	T	Protein of Unknown Function
182390	397698	400153	+	182390	99184	101644	+	182390	chromosome_6:397585-400661	Cre06.g252000.t1.1	T	Flagellar Associated Protein; found in the flagellar proteome [PMID: 15998802] Homolog to *H. sapiens* LZTFL1 (GenBank ID: AAH25988.1) Regulates the BBsome and may be a tumor suppressor
182389	401180	401794	-	182389	103532	104146	-	182389	chromosome_6:401009-402507	Cre06.g252050.t1.1	T	Protein of Unknown Function
294687	403265	412912	-	294687	105065	114642	-	522861	chromosome_6:403265-412912	Cre06.g252100.t1.1	T	Protein of Unknown Function
HDH1	414358	417535	-	HDH1	116631	119812	-	196269	chromosome_6:414358-418076	Cre06.g252150.t1.1	T	Haloacid dehalogenase-like hydrolase
TOC34	418285	421961	+	TOC34	120020	123826	+	187290	chromosome_6:418211-422361	Cre06.g252200.t1.1	T	Similar to the higher plant chloroplast outer envelope protein involved in protein import. Also called IAP34 and OEP34.
PDK1	432790	436759	-	PDK1	175950	179760	-	196270	chromosome_6:432547-437537	Cre06.g252300.t1.1	R	Putative pyruvate dehydrogenase kinase
CGL70	437858	440531	+	CGL70	180755	183180	+	196285	chromosome_6:437858-440531	Cre06.g252350.t1.1	R	Conserved protein of unknown function
NMDA1	441854	442918	-	NMDA1	199058	200122	-	182385	chromosome_6:441396-443425	Cre06.g252400.t1.1	R	Conserved protein of unknown function
DRG1	443823	446968	+	DRG1	200980	204132	+	167482	chromosome_6:443823-448018	Cre06.g252450.t1.2	R	Highly conserved GTP binding protein of unknown function. The developmentally regulated GTP-binding protein (DRG) subfamily is an uncharacterized member of the Obg family, an evolutionary branch of GTPase superfamily proteins.
DLA3	452915	456484	-	DLA3	288913	292126	-	187285	chromosome_6:452879-456728	Cre06.g252550.t1.2	R	Dihydrolipoamide acetyltransferase; E2 component of pyruvate dehydrogenase complex.
522872	464198	466309	+	522872	299691	302946	+	522872	chromosome_6:464198-466309	Cre06.g252600.t1.2	R	Protein of Unknown Function
LEU1S	470915	472682	-	LEU1S	317412	319190	-	126865	chromosome_6:470849-473034	Cre06.g252650.t1.1	R	Isopropylmalate dehydratase (EC 4.2.1.33); small subunit, involved in leucine biosynthesis.
SPL2	473720	476297	-	SPL2	321379	322919	-	196072	chromosome_6:473527-477186	Cre06.g252700.t1.1	R	Similar to splicing factor 3a, subunit 2
LPS1	495674	532121	-	LPS1	139144	171097	+	None	chromosome_6:495674-532121	None	R	Lipase domain protein. Gene model described under Genbank IDs:GU814014 & GU814015
522875	533457	536564	+	522875	135192	137781	-	522875	chromosome_6:533160-536919	Cre06.g252850.t1.1	R	Protein of Unknown Function. Contains partial eIF4B domain
PKY1	583784	594373	-	PKY1	260896	269217	+	522883	chromosome_6:583784-594373	Cre06.g253200.t1.2	R	Protein kinase.
MADS2	604587	607796	-	MADS2	251163	254600	+	142433	chromosome_6:604587-607796	Cre06.g253250.t1.2	R	Protein of unknown function; contains a MADS box; possible transcription factor.
UBCH1	608319	611626	-	UBCH1	247052	250463	+	182375	chromosome_6:608314-611993	Cre06.g253300.t1.1	R	Ubiquitin carboxyl-terminal hydrolase.
GCSH	612287	613424	+	GCSH	245616	246753	-	196067	chromosome_6:612078-614002	Cre06.g253350.t1.1	R	Glycine decarboxylase complex, H-protein; glycine cleavage system H protein; mitochondrial.
PR46a	613965	614790	+	PR46a	244254	245075	-	195751	chromosome_6:613946-615559		R	Conserved protein, probable ubiquitin-fold modifier 1 precursor. Contains a Ufm1 region.
PR46b	614566	616026	-	PR46b	242633	243969	+	24002	chromosome_6:614555-616550	Cre06.g253400.t1.2	R	Protein of Unknown Function
MT0618	617958	619173	+	MT0618	239874	241080	-	522889	chromosome_6:617958-619173	Cre06.g253459.t1.1	R	Protein of Unknown Function
155027	621009	621796	+	155027	237311	238039	-	155027	chromosome_6:621009-621796	Cre06.g253459.t1.1	R	Protein of Unknown Function
OTU2a	624393	626513	+	OTU2	232321	234447	-	155028	chromosome_6:624393-626513	Cre06.g253900.t1.1	R	Otubain domain putative protease.
HRGP1	786171	788252	-	HRGP1	329031	331111	-	522903	chromosome_6:785455-789453	Cre06.g254100.t1.2	R	Hydroxyproline-rich glycoprotein; possible cell wall component
UTP1	789569	794138	-	UTP1	332079	336649	-	195954	chromosome_6:789569-794291	Cre06.g254150.t1.1	R	Nucleolar protein, Component of the U3 processome. WD40 repeat protein.
MT0796	796333	796956	+	MT0796	338690	339306	+	None	chromosome_6:796333-796949	None	R	Protein of Unknown Function
MT0828	828295	828651	-	MT0828	345010	345366	-	167439	chromosome_6:828235-828651	Cre06.g254350.t1.2	C	Protein of Unknown Function
MT0829	829749	831363	-	MT0829	ND	ND	ND	522911	chromosome_6:828875-831773	Cre06.g254350.t1.2	C	Protein of Unknown Function
FUM1	832059	836131	+	FUM1	ND	ND	ND	195953	chromosome_6:831979-836593	Cre06.g254400.t1.2	C	Similarity to class I prokaryotic fumarase; predicted to be mitochondrial
FBX9	836668	838815	-	FBX9	ND	ND	ND	522913	chromosome_6:836605-839603	Cre06.g254450.t1.1	C	Protein of unknown function, containing cyclin-like F box, putative
522914	840544	843350	-	522914	ND	ND	ND	522914	chromosome_6:840255-843763	Cre06.g254500.t1.2	C	Protein of Unknown Function
522915	844306	844997	+	522915	ND	ND	ND	522915	chromosome_6:844204-845888	Cre06.g254550.t1.1	C	Protein of Unknown Function
294742	847211	852045	+	294742	ND	ND	ND	522916	chromosome_6:847211-852045	Cre06.g254600.t1.2	C	Protein of Unknown Function
522917	853364	855718	-	522917	ND	ND	ND	522917	chromosome_6:853364-855718	None	C	Protein of Unknown Function
522918	856079	857847	-	522918	ND	ND	ND	522918	chromosome_6:856005-859003	Cre06.g254650.t1.1	C	Protein of unknown function, containing zinc-finger domains
522919	859323	862240	+	522919	ND	ND	ND	522919	chromosome_6:859134-862538	Cre06.g254700.t1.2	C	Protein of Unknown Function
161193	862687	862992	-	161193	ND	ND	ND	161193	chromosome_6:862570-863757	Cre06.g254750.t1.1	C	Protein of Unknown Function
196073	863874	866894	-	196073	ND	ND	ND	196073	chromosome_6:863787-867288	Cre06.g254800.t1.1	C	SF3b4; Subunit of the splicing factor SF3B required for 'A' complex assembly formed by the stable binding of the U2 snRNP to the branch point sequence of the pre-mRNA. Also known as SAP49
522922	867943	876830	-	522922	ND	ND	ND	522922	chromosome_6:867755-878073	Cre06.g254850.t1.2	C	Protein of Unknown Function
SAD1	880383	897375	+	SAD1	ND	ND	ND	305997	chromosome_6:879988-897789	Cre06.g255000.t1.1	C	*MT-* agglutinin gene; sexual adhesion protein located on the flagellar surface of minus gametes; HRGP [PMID: 15659633, Genbank entry AY450929]
294752	906536	910391	-	294752	ND	ND	ND	522926	chromosome_6:906536-910391	Cre06.g255050.t1.1	C	Protein of Unknown Function
344092	910951	918193	+	344092	ND	ND	ND	344092	chromosome_6:910859-918193	Cre06.g255100.t1.1	C	Protein of Unknown Function
196063	921026	923570	+	196063	ND	ND	ND	196063	chromosome_6:920827-923570	Cre06.g255200.t1.1	C	Protein of unknown function; contains a (hydroxy)proline rich region
CGLD28	923951	930179	-	CGLD28	ND	ND	ND	187252	chromosome_6:923789-931283	Cre06.g255300.t1.1	C	Conserved protein
THI10	931565	933755	+	THI10	ND	ND	ND	126905	chromosome_6:931565-934443	Cre06.g255350.t1.1	C	hydroxyethylthiazole kinase (HMT Kinase, THIM), involved in thiamine biosynthesis. Mutants in this gene (THI10) are thiamine-requiring.
***MT+* Limited Genes**												
MTP0428	428272	432213	-	NA	NA	NA	NA	167487	chromosome_6:428272-432213	Cre06.g252250.t1.2	R	Protein of Unknown Function
FUS1	483650	487404	+	NA	NA	NA	NA	195935	chromosome_6:483650-488162	Cre06.g252750.t1.2	R	Glycoprotein localized to the membrane surface of the plus gamete mating structure; lacking in non-fusing fus1 mutants. The FUS1 gene exists only in the MT+ locus.
MTA5	547228	551384	-	NA	NA	NA	NA	522877	chromosome_6:546710-551888	Cre06.g252930.t1.1	R	Predicted Protein. Part of A region duplication in the mating type plus locus
MTA4	552115	559822	+	NA	NA	NA	NA	7933	chromosome_6:551994-560141	None	R	Predicted protein. Part of A region duplication in the mating type plus locus
psMTA2	561462	562582	-	NA	NA	NA	NA	305935	chromosome_6:561462-562582	None	R	Pseudogene. Original Hydroxyproline-rich glycoprotein interrupted by MTA1. Part of A region duplication in the mating type plus locus
MTA1	563451	563831	-	NA	NA	NA	NA	195674	chromosome_6:562980-564042	Cre06.g253000.t1.2	R	Protein of unknown function. mRNA is induced in plus gametes. Gene exists only in the mt+ locus [PMID: 11805055]
MTA3	567238	568053	+	NA	NA	NA	NA	195822	chromosome_6:567034-569191	None	R	Predicted Protein. Part of A region duplication in the mating type plus locus. [PMID: 11805055]
294708	577663	578485	-	NA	NA	NA	NA	294708	chromosome_6:577663-578485	Cre06.g253150.t1.1	R	Probable pseudogene due to 5' truncation. Part of A region duplication in the mating type plus locus
EZY2a	632370	633903	+	NA	NA	NA	NA	522890	chromosome_6:632370-633588	None	R	Zygotically expressed gene in 16 kb repeat region [PMID 11805055]
INT1a	648806	650537	-	NA	NA	NA	NA	522891	chromosome_6:648806-650537	Cre06.g253550.t1.1	R	Predicted Protein. Part of the "16kb Repeat Region"
EZY2b	658079	662115	+	NA	NA	NA	NA	522892	chromosome_6:657099-665573	Cre06.g253600.t1.2	R	Zygotically expressed gene in 16 kb repeat region [PMID 11805055]
INT1b	668047	670008	-	NA	NA	NA	NA	522893	chromosome_6:667970-670048	Cre06.g253650.t1.1	R	Predicted Protein. Part of the "16kb Repeat Region"
OTU2b	670189	671942	+	NA	NA	NA	NA	522894	chromosome_6:670189-671942	None	R	Otubain domain putative protease. Part of the "16kb Repeat Region"
EZY2c	676895	681300	+	NA	NA	NA	NA	522895	chromosome_6:676895-681300	Cre06.g253750.t1.1	R	Zygotically expressed gene in 16 kb repeat region [PMID 11805055]
EZY2d	724806	729214	+	NA	NA	NA	NA	522896	chromosome_6:724806-729214	Cre06.g253950.t1.1	R	Zygotically expressed gene in 16 kb repeat region [PMID 11805055]
INT1c	733883	735844	-	NA	NA	NA	NA	522897	chromosome_6:733883-735844	None	R	Predicted Protein. Part of the "16kb Repeat Region"
OTU2c	736063	737819	+	NA	NA	NA	NA	522898	chromosome_6:736063-737819	None	R	Otubain domain putative protease. Part of the "16kb Repeat Region"
EZY2e	749135	752889	+	NA	NA	NA	NA	522899	chromosome_6:749135-752889	None	R	Zygotically expressed gene in 16 kb repeat region [PMID 11805055]
EZY2f	779676	780628	+	NA	NA	NA	NA	522902	chromosome_6:779574-782608	None	R	Zygotically expressed gene in 16 kb repeat region [PMID 11805055]
SRLc	798573	799852	-	NA	NA	NA	NA	None	chromosome_6:798573-799852	None	R	Predicted Protein. Described under Genbank ID:GU814014
SRLb	815243	821387	-	NA	NA	NA	NA	None	chromosome_6:812387-815243	None	R	Predicted Protein. Described under Genbank ID:GU814014
SRLa	821696	824156	-	NA	NA	NA	NA	None	chromosome_6:821696-824156	None	R	Predicted Protein. Described under Genbank ID:GU814014
MT- Limited Genes												
NA	NA	NA	NA	psEZY2	226695	229067	-	NA	NA	NA	R	Pseudogene copy of EZY2
NA	NA	NA	NA	MID	286502	287123	+	NA	NA	NA	R	Minus Dominance. Putative RWP-RK domain transcription factor that specifies minus mating type [PMID 17435233]
NA	NA	NA	NA	MTD1	308874	311600	-	NA	NA	NA	R	Protein that contributes to minus mating type differentiation [PMID 17435233].

Sequences and annotation of the *MT+* and *MT-* locus haplotypes are described in [11,19] and available in Genbank under accession numbers GU814014 and GU814015
+: Augustus v5 Model ID's begin with "5"
*: *MT-* coordinates are based upon the Genbank entry noted above. The Augustus v10.2 Model IDs were determined using the Algal Functional Annotation Tool at the following URL: http://pathways.mcdb.ucla.edu/chlamy/id_conversion.html. The start and stop locations of each of the *MT+* gene models are based on the v4 JGI genome assembly. NA: not applicable. ND: Not determined
