# Supplementary material for: Species and Population Level Molecular Profiling Reveals Cryptic Recombination and Emergent Asymmetry in the Dimorphic Mating Locus of C. reinhardtii
Source: PLoS Genet. 2013 Aug 29;9(8):e1003724. doi: 10.1371/journal.pgen.1003724 (PMC3757049; doi:10.1371/journal.pgen.1003724)
Supplement: Table S5 — Summary of expression data for mating locus genes. JGI EST: Number of ESTs mapped to the gene model on the Phytozome browser. + one or more EST matches. − no EST matches. Probes from previous study [19] were matched to their overlapping gene model(s) in the JGI V4 C. reinhardtii genome assembly. #, Probe 65 was in the intergenic region between LEU1S and 522872 and most likely detected RNA from a transposable element. Expression stage is abbreviated as Veg, vegetative; Gam, gametic; Zyg, zygotic; all stages, All; ND, not detected; NA, not available 454: Number of 454 cDNA sequences that map to the gene model on the UCLA MCDB/MBI Genome Browser http://genomes.mcdb.ucla.edu/Cre454/project.html. + one or more 454 matches. − no 454 matches. All 454 sequences corresponding to duplicated MT+ genes in the SRL and MTA regions were realigned to the MT+ and autosomal gene copies, and polymorphisms were used to distinguish the origin of the transcript. Positive evidence of a transcript is indicated only when genomic origin could be determined. JGI v4 PID: JGI C. reinhardtii v4 Protein ID (if available) for the listed gene model. a from [19]. (PDF) [file pgen.1003724.s011.pdf]

TABLE S5
Summary of Expression Data for Mating Locus Genes.

Shared Genes

Gene	JGI EST	JGI v4 ID	Northern Blot Probe[a]	Stage of Expression[a]	qRT-PCR	454
NIC7	-	522848	Nic7	Veg		+
SPS1	-	206416				+
SPP3	-	196107	Pr6	Zyg		+
EIF5Bb	+	206391	Pr7	All		+
PTC1	+	196076				+
182394	+	182394	Pr9	All		+
97782	+	97782				+
RFC4	+	195837				+
182392	+	182392	BG860484	Veg		+
ALB3	+	187295	Pr11 (Ac29)	Veg		+
PSF2	-	522858				+
182390	+	182390				+
182389	+	182389				+
294687	-	294687				+
HDH1	+	196269				+
TOC34	+	187290				+
PDK1	+	196270	Pdk	Veg		+
CGL70	+	196285				+
NMDA1	+	182385	Pr43*	All	All	+
DRG1	-	167482	Pr44*	All		+
DLA3	+	187285			All	+
522872	-	522872	Pr65#	All		+
LEU1S	+	126865	Pr65#	All		+
SPL2	+	196072				+
LPS1	-	NA	Pr105	Veg		+
522875	+	522875				+
PKY1	+	294709			All	+
MADS2	-	142433			Minus Veg/Gam	+
UBCH1	+	182375				+
GCSH	+	196067	GcdH	Veg		+
PR46a	+	195751	Pr46a	Veg		+
PR46b	+	24002	Pr46b			+
MT0618	-	NA				+
155027	-	155027				+
OTU2a		NA			Gam	+
HRGP1	-	522903	Pr71	Gam	Gam	+
UTP1	+	195954				+
MT0796	-	NA	Pr72	Zyg	Zyg	+
MT0828	+	167439	Pr74	Zyg	Zyg	+
MT0829	-	522911				+
FUM1	+	195953				+
FBX9	+	522913				+
522914	-	522914				+
522915	+	522915				+
294742	+	294742				-
522917	-	522917				+
522918	+	522918				+
522919	-	522919				+
161193	+	161193				+
196073	+	196073	Pr81	Veg		+
522922	+	522922				+
SAD1	+	305997	Sad1	Minus Gam	Minus Gam	+
294752	+	294752				+
344092	+	344092				+
196063	+	196063				+

MT- Limited Genes

Gene	JGI EST	JGI v4 ID	Northern Blot Probe[a]	Stage of Expression[a]	qRT-PCR	454
MID	NA	NA		Minus Gam	Gam	
MTD	NA	NA		Minus Gam		

MT+ Limited Genes

Gene	JGI EST	JGI v4 ID	Northern Blot Probe[a]	Stage of Expression[a]	qRT-PCR	454
MTP0428	-	167487			Zyg	+
FUS1	-	195935	Fus1	Plus Gam	Gam	+
MTA5	-	522877				-
MTA4	-	7933			Zyg	+
psMTA2	-	305935				-
MTA1	-	195674		Gam		+
MTA3	+	195822				+
294708	-	294708	Pr120*	Gam		-
EZY2a	-	522890			Zyg	+
INT1a	-	522891			ND	-
EZY2b	+	522892			Zyg	+
INT1b	-	522893			ND	-
OTU2b	-	522894			Gam	+
EZY2c	-	NA			Zyg	+
EZY2d	-	NA				+
INT1c	-	522897				-
OTU2c	+	522898			Gam	+
Ezy2e	-	522899				+
Ezy2f	+	522902				+
SRL1a	-	NA				-
SRL1b	-	NA			Gam	+
SRL1c	-	NA				-

JGI EST: Number of ESTs mapped to the gene model on the Phytozome browser. +, one or more EST matches. -, no EST matches.
Probes from previous study [18] were matched to their overlapping gene model(s) in the JGI V4 C. reinhardtii genome assembly. #, Probe 65 was in the intergenic region between LEU1S and 522872 and most likely detected RNA from a transposable element. Expression stage is abbreviated as Veg, vegetative; Gam, gametic; Zyg, zygotic; all stages, All; ND, not detected; NA, not available
454: Number of 454 cDNA sequences that map to the gene model on the UCLA MCDB/MBI Genome Browser (http://genomes.mcdb.ucla.edu/Cre454/project.html) +, one or more 454 matches. -, no 454 matches. All 454 sequences corresponding to duplicated MT+ genes in the SRL and MTA regions were realigned to the MT+ and autosomal gene copies, and polymorphisms were used to distinguish the origin of the transcript. Positive evidence of a transcript is only indicated when its genomic origin can be determined.
JGI v4 PID: JGI C. reinhardtii v4 Protein ID, where available, for the listed gene model. a. [19]
