# Supplementary material for: Species and Population Level Molecular Profiling Reveals Cryptic Recombination and Emergent Asymmetry in the Dimorphic Mating Locus of C. reinhardtii
Source: PLoS Genet. 2013 Aug 29;9(8):e1003724. doi: 10.1371/journal.pgen.1003724 (PMC3757049; doi:10.1371/journal.pgen.1003724)
Supplement: Table S6 — Chlamydomonas reinhardtii strains and DNA sequences used for population genetic studies. Chlamydomonas Resource Center (http://chlamycollection.org/) strain numbers are listed along with common laboratory names for selected strains. Geographic origins are abbreviated as follows: FL, Florida; MA, Massachusetts; MN, Minnesota; NC, North Carolina; PA, Pennsylvania; QC, Quebec, Canada. Genbank accession numbers are listed for genes from each isolate. a Data from [37]. (PDF) [file pgen.1003724.s012.pdf]

TABLE S6
***C. reinhardtii* strains and DNA sequences used for population genetic studies**

Strain ID	Alternative name	Geographic Origin	Mating type	Genbank accession numbers											
				MID	*MTA1*	*GP1*	*mito*DNA	*PDK1*	*PR46b*	*SAD1*	*SPP3*	*CBLPa*[a]	*MAT3a*[a]	*IDA5/ACTIN*[a]	*YPT4*[a]
CC-277	cw15-2	MA	MT+									EU306630	EU306632	D50838	U13167
CC-620	R3 (137c)	MA	MT+		AF417571	GU002580	GU002593	GU002606	GU002619	GU002632	GU002645				
CC-1373	*C. smithii*	MA	MT+		DQ364190	GU002581	GU002594	GU002607	GU002620	GU002633	GU002646	EU306625	EU306633	U70571	U55911
CC-2343	Jarvik 124	PA	MT+		DQ364191	GU002582	GU002595	GU002608	GU002621	GU002634	GU002647	EU306628	EU306636	U70561	U55889
CC-2344	Jarvik 356	FL	MT+		DQ364192	GU002583	GU002596	GU002609	GU002622	GU002635	GU002648	EU306629	EU306637	U70562	U55891
CC-2932	Harris 10	NC	MT+		DQ364193	GU002584	GU002597	GU002610	GU002623	GU002636	GU002649				
CC-2936	-	QC	MT+		DQ364194	GU002585	GU002598	GU002611	GU002624	GU002637	GU002650				
CC-2937	-	QC	MT+		DQ364195	GU002586	GU002599	GU002612	GU002625	GU002638	GU002651				
CC-621	NO (137c)	MA	MT-	U92071		GU002587	GU002600	GU002613	GU002626	GU002639	GU002652				
CC-1952	*C. grossii,* S1C5	MN	MT-	DQ355810		GU002588	GU002601	GU002614	GU002627	GU002640	GU002653	EU306626	EU306634	U70563	U55893
CC-2342	Jarvik 6	PA	MT-	DQ355811		GU002589	GU002602	GU002615	GU002628	GU002641	GU002654	EU306627	EU306635	U70569	U55905
CC-2931	Harris 6	NC	MT-	DQ355812		GU002590	GU002603	GU002616	GU002629	GU002642	GU002655	EU306624	EU306631	U70568	U55901
CC-2935	-	QC	MT-	DQ355808		GU002591	GU002604	GU002617	GU002630	GU002643	GU002656				
CC-2938	-	QC	MT-	DQ355809		GU002592	GU002605	GU002618	GU002631	GU002644	GU002657				

Chlamydomonas Resource Center (http://chlamycollection.org/) strain numbers are listed along with common laboratory names for selected strains. Geographic origins are abbreviated as follows: FL, Florida; MA, Massachusetts; MN, Minnesota; NC, North Carolina; PA, Pennsylvania; QC, Quebec, Canada. Genbank accession numbers are listed for genes in each isolate. a. Data from [37]
