# Supplementary material for: Species and Population Level Molecular Profiling Reveals Cryptic Recombination and Emergent Asymmetry in the Dimorphic Mating Locus of C. reinhardtii
Source: PLoS Genet. 2013 Aug 29;9(8):e1003724. doi: 10.1371/journal.pgen.1003724 (PMC3757049; doi:10.1371/journal.pgen.1003724)
Supplement: Table S7 — Population data and haplotype differentiation for mating locus and autosomal genes. na not applicable. 1. Number of MT+ and MT− strains analyzed for each gene. 2. Total number of silent sites (non-coding and synonymous) 3. Number of segregating silent sites. 4. Polymorphism rate for silent sites. Standard deviation in parentheses. 5. Tajima's D statistic calculated for silent substitutions. Significant value (p<.05) is in bold. nd indicates not done for groups with less than 4 sequences. 6. dxy Average pairwise substitution rate between MT+ and MT− isolates with Jukes-Cantor correction. 7. dA residual difference between MT+ and MT− isolates when corrected for within-population divergence. Standard deviation in parentheses. Bold values are samples with dA scores outside of one standard deviation from the null value of zero. 8. Population differentiation between MT+ and MT− isolates. (PDF) [file pgen.1003724.s013.pdf]

TABLE S7

Population Genetic Data for *MT* and autosomal genes

	no. sequences[1]		no. silent sites[2]	S sil[3]			π sil[4]			Tajima's D sil[5]			d_{XY}[6]	d_A[7]	F_{ST}[8]
	***MT*+**	***MT*-**		**total**	***MT*+**	***MT*-**	**total**	***MT*+**	***MT*-**	**total**	***MT*+**	***MT*-**			
**R Domain sex-limited**															
MTA1	7	na	406	4	4	na	na	0.00422 (0.00111)	na	0.239	0.239	na	na	na	na
MID	na	6	330	9	na	9	na	na	0.01111 (0.00338)	-0.415	na	-0.415	na	na	na
**R Domain shared**															
PR46	7	6	504	17	1	8	0.01597 (0.00180)	0.00113 (0.00024)	0.00608 (0.00194)	**1.984**	1.342	-0.735	0.02083 (0.00709)	**0.01773** (0.00714)	0.85
PDK1	7	6	675	40	2	29	0.02330 (0.00342)	0.00127 (0.00032)	0.01877 (0.00451)	0.848	0.206	-0.228	0.02622 (0.00783)	**0.018996** (0.00802)	0.7213
**C/T domain shared**															
SPP3	7	6	562	73	57	61	0.04558 (0.00420)	0.04465 (0.00703)	0.05184 (0.00736)	0.146	0.201	0.278	0.03256 (0.01029)	-0.00344 (0.01085)	-0.10563
MAT3	4	3	690	40	30	6	0.02374 (0.00343)	0.02464 (0.00588)	0.00580 (0.00232)	-0.011	0.231	nd	0.01993 (0.00738)	**0.00904 (0.00777)**	0.45217
SAD1	7	6	317	41	28	35	0.04384 (0.00696)	0.03185 (0.00872)	0.05005 (0.01618)	0.25	-0.664	0.0189	0.02061 (0.00655)	0.00334 (0.00718)	0.16461
**Autosomal**															
GP1	7	6	420	37	35	20	0.02506 (0.00525)	0.03129 (0.00782)	0.02079 (0.00444)	-0.721	-0.745	-0.018	0.01584 (0.00564)	-0.00179 (0.00565)	-0.11345
IDA5/Actin	4	3	214	21	15	12	0.03560 (0.00538)	0.03816 (0.00715)	0.03738 (0.01498)	-0.625	-0.019	nd	0.03481 (0.01454)	-0.00411 (0.01626)	-0.11494
CBLP	4	3	681	78	62	50	0.04993 (0.00632)	0.04968 (0.01427)	0.04895 (0.01595)	0.24	-0.161	nd	0.03522 (0.01564)	0.00069 (0.01765)	0.01946
YPT4	4	3	790	47	26	31	0.02266 (0.00450)	0.01688 (0.00486)	0.02616 (0.01156)	-0.385	-0.617	nd	0.02517 (0.0109)	0.00320 (0.01217)	0.12821

Notes: na not applicable. 1. Number of *MT*+ and *MT*- strains analyzed for each gene. 2. Total number of silent sites (non-coding and synonymous) 3. Number of segregating silent sites. 4. Polymorphism rate for silent sites. Standard deviation in parentheses. 5. Tajima's D statistic calculated for silent substitutions. Significant value ($p<.05$) is in bold. nd indicates not done for groups with less than 4 sequences. 6. d_{xy} Average pairwise substitution rate between *MT*+ and *MT*- isolates with Jukes-Cantor correction. 7. d_A residual difference between *MT*+ and *MT*- isolates when corrected for within-population divergence. Standard deviation in parentheses. Bold values are samples with dA scores outside of one standard deviation from the null value of zero. 8. Population differentiation between *MT*+ and *MT*- isolates.
