# Supplementary material for: Species and Population Level Molecular Profiling Reveals Cryptic Recombination and Emergent Asymmetry in the Dimorphic Mating Locus of C. reinhardtii
Source: PLoS Genet. 2013 Aug 29;9(8):e1003724. doi: 10.1371/journal.pgen.1003724 (PMC3757049; doi:10.1371/journal.pgen.1003724)
Supplement: Table S8 — Recombination data for MT+ homozygous cross. Parental strains K33 and CC-2344 (both MT+), were crossed and progeny that showed recombination between NIC7 and THI10 were scored for additional markers in the indicated genes. The first 8 markers are in MT and listed in the order they occur on chromosome 6. MMP1, YPT4 and GP1 are unlinked to MT and were used as controls to show independent assortment of autosomal markers in the cross. Nic and Thi columns indicate auxotrophy (−) or prototrophy (+) for nicotinamide and thiamine respectively. (PDF) [file pgen.1003724.s014.pdf]

TABLE S8
Recombination data for *MT*+ homozygous cross.

	Nic	NIC7	PDK1	MTA1	PR46a	SAD1	Thi	MAT3	MMP1	YPT4	GP1
					**Parents**						
K33	–	K33	K33	K33	K33	K33	–	K33	K33	K33	K33
CC–2344	+	356	356	356	356	356	+	356	356	356	356
					**Progeny**						
BA17	+	356	nd	nd	nd	356	–	K33	K33	356	K33
BA23	–	K33	K33	356	nd	356	+	356	356	K33	K33
BA25	–	K33	nd	nd	nd	K33	+	356	K33	K33	K33
BB22	+	356	356	356	K33	K33	–	K33	356	K33	356
BB25	–	K33	nd	nd	nd	K33	+	356	K33	K33	K33
BC10	–	K33	nd	nd	nd	K33	+	356	356	K33	356
BC29	+	356	nd	nd	nd	356	–	K33	356	K33	356
BC43	–	K33	nd	nd	nd	K33	+	356	356	K33	356
BD23	–	K33	K33	K33	356	356	+	356	K33	K33	K33
BD25	–	K33	K33	K33	K33	356	+	356	356	K33	356
BF27	+	356	nd	nd	nd	356	–	K33	356	K33	K33
BF30	–	K33	nd	nd	nd	K33	+	356	356	356	K33
BF39	+	356	356	K33	nd	K33	–	K33	356	356	K33
BG7	+	356	356	K33	nd	K33	–	K33	K33	356	356
BH14	+	356	nd	nd	nd	356	–	K33	K33	K33	356
BH29	–	K33	nd	nd	nd	K33	+	356	K33	356	356
BH39	–	K33	nd	nd	nd	K33	+	356	356	356	K33

Parental strains K33 and CC-2344 (both *MT*+), were crossed and progeny that showed recombination between NIC7 and THI10 were scored for additional markers in the indicated genes. The first 8 markers are in *MT* and listed in the order they occur on chromosome 6. MMP1, YPT4 and GP1 are unlinked to *MT* and were used as controls to show independent assortment of autosomal markers in the cross. Nic and Thi represent auxotrophy (-) or prototrophy (+) for nicotinamide or thiamine respectively.
