# Supplementary material for: Species and Population Level Molecular Profiling Reveals Cryptic Recombination and Emergent Asymmetry in the Dimorphic Mating Locus of C. reinhardtii
Source: PLoS Genet. 2013 Aug 29;9(8):e1003724. doi: 10.1371/journal.pgen.1003724 (PMC3757049; doi:10.1371/journal.pgen.1003724)
Supplement: Table S9 — Recombination data for MT− homozygous cross. 1. recombinant progeny/total progeny. 2. Expected recombinants for MAT3-PDK1 and for MID-NIC7 are based on the genome-wide average of ∼1 cM/100 kb. For 4121-MT and GAR1-GSAT the expected value is based on previous data [49]. (PDF) [file pgen.1003724.s015.pdf]

TABLE S9
Recombination data for *MT-* x *MT-* progeny

Linked Markers	Chromosome	Recombinants[1]		Measured cM
		expected[2]	observed	
MID-NIC7	VI (*MT*)	14/600	0/600	0
MAT3-PDK1	VI (*MT*)	3/146	0/146	0
4121-MT	VI	27/96	26/96	27
GAR1-GSAT	III	20/96	20/96	21

1. recombinant progeny/total progeny. 2. Expected recombinants for *MID-NIC7* and *MAT3-PDK1* is based on the genome-wide average of ~1 cM/100 kb. For *4121-MT* (mating type) and *GAR1-GSAT* the expected value is based on published data [49].
