# Supplementary material for: Species and Population Level Molecular Profiling Reveals Cryptic Recombination and Emergent Asymmetry in the Dimorphic Mating Locus of C. reinhardtii
Source: PLoS Genet. 2013 Aug 29;9(8):e1003724. doi: 10.1371/journal.pgen.1003724 (PMC3757049; doi:10.1371/journal.pgen.1003724)
Supplement: Table S10 — List of oligonucleotides used in this study. a primers derived from [78]. b primers derived from [79]. c primers derived from [73]. (PDF) [file pgen.1003724.s016.pdf]

TABLE S10
List of oligonucleotides used in this study

Primer Name	Sequence	Gene	Description	Purpose
Anchored Oligo dT20	TTTTTTTTTTTTTTTTTTTTVN		Anchored olido dT primer	1st strand cDNA synthesis primer
MADS2.DelCheck.f1	GAACGCCGTCGTAAGCGAGTTCT	MADS2	MADS2Minus Promter Region. Flanks 17bp deletion in Plus. 120bp pdt w/17bp present. (in MT-)	MADS Upstream Deletion
MADS2.DelCheck.r1	TGTACCATCAAACGGGAAGCCGC	MADS2	MADS2Minus Promter Region. Flanks 17bp deletion in Plus. 120bp pdt w/17bp present. (in MT-)	MADS Upstream Deletion
MTP0428.Auto.RT.f1	GAATGAGGATCATCTGGCAAGTGAGGAG	294656	217bp product. Specific to the autosomal, not MT, copy.	quantitative RT-PCR
MTP0428.Auto.RT.r1	CTCCGCTACCGAGGCCACCATTAA	294656	217bp product. Specific to the autosomal, not MT, copy.	quantitative RT-PCR
DLA3.f1	CGGCAAGGCATCTTAAACCCTGCAT	DLA3	160bp product	quantitative RT-PCR
DLA3.r1	GTGAAAGCGTGGCATGTCACTGGAG	DLA3	160bp product	quantitative RT-PCR
EZY2a.RT.f1	GCGACAGGAGCTTCGAGGAGTGG	EZY2abcde	177bp product	quantitative RT-PCR
EZY2a.RT.r1	TTGTTGTTGGTGGTGGTGATGGTGG	EZY2abcde	177bp product	quantitative RT-PCR
FUS1.RT.f1	GCTTACGAGGCTGTGACGCTTTTTG	FUS1	212bp product	quantitative RT-PCR
FUS1.RT.r1	TTTCCTAGCCGCTTGCGTTCGTTAC	FUS1	212bp product	quantitative RT-PCR
HRGP1.RT.f1	TTCGAAGGGACAATGGGTTTGACGC	HRGP	167bp pdt	quantitative RT-PCR
HRGP1.RT.r1	GCGTTGGTTACAAAAAGGACAGCGG	HRGP	167bp pdt	quantitative RT-PCR
Int.RT.f1	CGTCTTCAGCGTGACCTACAACACC	Int1ab	235bp pdt	quantitative RT-PCR
Int.RT.r1	ATGACGGGTCATCTCCAGAGCGG	Int1ab	235bp pdt	quantitative RT-PCR
MADS2.f1	CCGGCCTCAACGGGGAAAAGTATTG	MADS2	181bp product	quantitative RT-PCR
MADS2.r1	CGTGAGCAACAGTAGCGTCATGCAG	MADS2	181bp product	quantitative RT-PCR
Mid.RT.f2	GAGCCCAGGACGAGATCCTTTGAGG	MID	162bp product	quantitative RT-PCR
Mid.RT.r2	TCGGCCAGAACCTTTCATAGGGTGG	MID	162bp product	quantitative RT-PCR
M-OTU2a.RT.f1	GGAGCGCCTCTTCAACAGCGC	MT- OTU2a	274bp product. Specifc to the MT- copy	quantitative RT-PCR
M-OTU2a.RT.r1	GGCTCCCACCGCACTGCCT	MT- OTU2a	274bp product. Specifc to the MT- copy	quantitative RT-PCR
P-OTU2abc.RT.f1	ACCGCCTGGTTCAAGTGCCCC	MT+ OTU2abc	357bp product. Specific to the MT+ copy	quantitative RT-PCR
P-OTU2abc.RT.r1	GGCTCCCACCGCACTGCCC	MT+ OTU2abc	357bp product. Specific to the MT+ copy	quantitative RT-PCR
MT0796.RT.f2	GCCCTTCGATTACTCATTCAGCAGGC	MT0796	182bp product	quantitative RT-PCR
MT0796.RT.r2	CTCCACACCATCCAAGCATGCACC	MT0796	182bp product	quantitative RT-PCR
MT0828.RT.f2	TACATCTGACTACGAGCCCACGACG	MT0828	181bp product	quantitative RT-PCR
MT0828.RT.r2	CCACGCATTTGAAGGCCCGC	MT0828	181bp product	quantitative RT-PCR
MTP0428.RT.f1	GACCAGCCGGCAGGTTCCA	MTP0428	208 bp product. Specific to the MT copy and none of the autosomal varients	quantitative RT-PCR
MTP0428.RT.r1	GCTCCGCTACCGAGATCACCATTAG	MTP0428	208 bp product. Specific to the MT copy and none of the autosomal varients	quantitative RT-PCR
NMDA1.RT.f1	CATAGTGCTGCTGGCCTTTGGACTG	NMDA1	217bp pdt	quantitative RT-PCR
NMDA1.RT.r1	GATGTACACACTGAACACCACCGCC	NMDA1	217bp pdt	quantitative RT-PCR
OTU2.Uni.RT.f2	TCTACACTCCCTTCATTCCCGGCTG	OTU2abc	115bp product	quantitative RT-PCR
OTU2.Uni.RT.r2	ATGATCTGCAGCTGCTCAACCTCCA	OTU2abc	115bp product	quantitative RT-PCR
PKY1.f1	CACCTCAAGCGTTCAGTGGGTTAGC	PKY1	141bp product	quantitative RT-PCR
PKY1.r1	CAGTGCTGTGCGTTGGAGTGGTTTA	PKY1	141bp product	quantitative RT-PCR
SadPlus.RT.f1	ACGCCATAATGGACGAGATGGAGGG	SAD1	180bp product	quantitative RT-PCR
SadPlus.RT.f2	TCTGGACGTGTCGACGATGAGACTG	SAD1	180bp product	quantitative RT-PCR
SRL1b.454Supp.f1	CGCATGATTGAGGATGCGGCGA	SRL1b	237bp product	quantitative RT-PCR
SRL1b.454Supp.r1	CGCTTGCCGTGAACGTTGCAATTGC	SRL1b	237bp product	quantitative RT-PCR
GBLP-1	GAGTCCAACTACGGCTACGC	GBLP	Control RT PCR Primers for *C. reinhardtii.*	quantitative RT-PCR
GBLP-2	AGCTTGCAGTTGGTCAGGTT	GBLP	Control RT PCR Primers for *C. reinhardtii.*	quantitative RT-PCR
18SrRNA-1	ATCTGCGAAAGCATTTGCCA	18S[c]	18s rDNA	quantitative RT-PCR
18SrRNA-2	CGGCATCGTTTATGGTTGAGAC	18S[c]	18s rDNA	quantitative RT-PCR
MTA4.f1	GCACGTGGGCCTTCTTCGCG	MTA4	290bp product. Specific to the MT copy	quantitative RT-PCR
MTA4.r1	GCCCGAGCCCTTGTTGTACACG	MTA4	290bp product. Specific to the MT copy	quantitative RT-PCR
**Primers used in the population study**				
mid7	AGAGCGCTTTCCATACC	MID	MID	Population Study
mid8	CACAGTTGCTAGTGCTC	MID	MID	Population Study
A1	CGTCGTCAGTACTCCCA	MTA1	The 761 bp PCR product is digested by MseI into 453, 259 and 49 bp fragments in K33; 453 and 308 in CC-2344.	Population Study
A2	CCACTAGCTAACGTCCC	MTA1		Population Study
BD12	TGTGAGTCCAAGTGCTGG	PDK1	see below	Population Study
BD15	GGAGGCATGCAAAGCATTGC	PDK1		Population Study
PO2-17	CTCAGAACTGCGCCTGATTC	PR46a	The 756 bp PCR product was sequenced directly using primer PO2-18 to identify the single polymorphism.	Population Study
PO2-18	CCATCAACCATGAGTCCATG	PR46a		Population Study
B5-9	GACGGTCTTGTTCTCCGC	SPP3		Population Study
B5-14	GTGCAAGGTCATGGGTAACG	SPP3		Population Study
AG77	AGCCAGCCGTTCAAGCAGTC	SAD1	The ~1210 bp PCR product is digested by PvuII into 512, 435, 225, 31 and 9 bp fragments in K33; 438, 368, 225, 144 and 31 in CC-2344. Product is within the agglutinin head domain, not the repetitive shaft domain.	Population Study
AG78	CCACCTCGCTCCAGTCATCC	SAD1		Population Study
gp8	GTGCGTCATACTAAGCTG	GP1	The 842 bp PCR product is digested by BspHI into 638 and 204 bp fragments in K33. CC-2344 is not cut.	Population Study
gp11	GGCCGTCAAGCTGGTTTG	GP1		Population Study
mito1	CCAAGTAACTACCCACATAG	mitDNA	mitDNA	Population Study
mito2	CACCTTATGACTCACGCTAG	mitDNA	mitDNA	Population Study
**Additional primers used in the genetic analysis**				
NIC13	CATCAACACCTCGCTGCG	NIC7	The 250 bp PCR product is digested by PvuII into 165 and 85 bp fragments in CC-2344. K33 is not cut.	Recombination scoring
NIC14	CTGATGCTGTCCACCGTG	NIC7		Recombination scoring
BD15	GGAGGCATGCAAAGCATTGC	PDK1	The 710 bp PCR product is digested by HpyCH4IV into 492 and 218 bp fragments in K33; 397, 218 and 95 in CC-2344.	Recombination scoring
BD22	CTTCAGGTCGTCTCCAGC	PDK1		Recombination scoring
mat3-34	CCTTGACGTCTGCTCATCAAGC	MAT3	The 811 bp PCR product is digested by AvaII into 520 and 291 bp fragments in K33. CC-2344 is not cut.	Recombination scoring
mat3-35	CAAACAGCACCCCAACAAGC	MAT3		Recombination scoring
gle1	CGTAGTGTGCATGCTGCTGCAGTA	MMP1[a]	The 394 bp PCR product is digested by MfeI into 300 and 94 bp fragments in K33. CC-2344 is not cut.	Recombination scoring
gle2	TCCCATAGGTGTTACAACCATATCA	MMP1[a]		Recombination scoring
ypt4/6 5'	AGCACVGCNCACAACGTNGAGGA	YPT4[b]	The 535 bp PCR product is digested by RsaI into 254, 166 and 115 bp fragments in K33; 369 and 166 in CC-2344.	Recombination scoring
ypt4/6 3'	AGTCTCCTTNGCBGTGTTRATGAA	YPT4[b]		Recombination scoring
PDK1_MfeIF2	GGTACCTTTGCCAGACTCGTCATA	PDK1	The 569 bp PCR product is digested by MfeI into 152 and 417 bp fragments in B32. CC1952 is not cut.	Recombination scoring
PDK1_MfeIR2	GTGTGCGTAAGAGCGAGTACATATC	PDK1		Recombination scoring
MAT3_BciVIF1	ATAACCCGTATGGCTTGCAG	MAT3	The 597 bp PCR product is digested by BciVI into 381 and 216 bp fragments in B32. The CC1952 amplicon is not cut.	Recombination scoring
MAT3_BciVIR1	CCTGTCAGGACTCACAAGCA	MAT3		Recombination scoring
4121_R1	CCCCTTGTGTATCGGTATGG	4121[d]	B32 and CC1952 yield 290 bp and 310 bp PCR products respectively.	Recombination scoring
4121_F1	GCGACACGACCTCTATCACA	4121[d]		Recombination scoring
MethSyn_R	GCAATGCGTTGGGTTACAAGCAGC	GAR1[d]		Recombination scoring
MethSyn_F2	GCGAGCGGTACCGACTAGGCAGA	GAR1[d]	B32 and CC1952 yield 339 and 179 bp PCR prooducts respectively.	Recombination scoring
MethSyn_F3	GCTGAATTGTGTACGGTGCACACGG	GAR1[d]		Recombination scoring
GSAT_R	GAGGGTGCAATCAGAGCCCCCTTG	GSAT[d]		Recombination scoring
GSAT_F2	CGCGTGCACAGCTTGCAGCAAA	GSAT[d]	B32 and CC1952 yield 561 and 389 bp PCR products respectively.	Recombination scoring
GSAT_F3	CGGGCGGTGCCTGGTTCTTCG	GSAT[d]		Recombination scoring

a. primers derived from[78]
b. primers derived from[79]
c. primers derived from[73]
d. primers derived from[49]
